# The nuclear import receptor Kapβ2 modifies neurotoxicity mediated by poly(GR) in C9orf72-linked ALS/FTD

**DOI:** 10.1038/s42003-024-06071-2

**Published:** 2024-03-28

**Authors:** M. E. Cicardi, V. Kankate, S. Sriramoji, K. Krishnamurthy, S. S. Markandaiah, B. M. Verdone, A. Girdhar, A. Nelson, L. B. Rivas, A. Boehringer, A. R. Haeusler, P. Pasinelli, L. Guo, D. Trotti

**Affiliations:** 1 Weinberg ALS Center, Vickie and Jack Farber Institute for Neuroscience, Department of Neuroscience, Thomas Jefferson University, Philadelphia, PA USA; 2 Department of Biochemistry and Molecular Biology, Thomas Jefferson University, Philadelphia, PA USA

**Keywords:** Amyotrophic lateral sclerosis, Cell death in the nervous system

## Abstract

Expanded intronic G_4_C_2_ repeats in the *C9ORF72* gene cause amyotrophic lateral sclerosis (ALS) and frontotemporal dementia (FTD). These intronic repeats are translated through a non-AUG-dependent mechanism into five different dipeptide repeat proteins (DPRs), including poly-glycine-arginine (GR), which is aggregation-prone and neurotoxic. Here, we report that Kapβ2 and GR interact, co-aggregating, in cultured neurons in-vitro and CNS tissue in-vivo. Importantly, this interaction significantly decreased the risk of death of cultured GR-expressing neurons. Downregulation of Kapβ2 is detrimental to their survival, whereas increased Kapβ2 levels mitigated GR-mediated neurotoxicity. As expected, GR-expressing neurons displayed TDP-43 nuclear loss. Raising Kapβ2 levels did not restore TDP-43 into the nucleus, nor did alter the dynamic properties of GR aggregates. Overall, our findings support the design of therapeutic strategies aimed at up-regulating Kapβ2 expression levels as a potential new avenue for contrasting neurodegeneration in C9orf72-ALS/FTD.

## Abstract (web summary)

Expanded intronic G_4_C_2_ repeats in C9orf72 cause ALS/FTD. GR dipeptide repeats produced in C9orf72 ALS/FTD co-aggregate with Kap2 in neurons, affecting survival. Upregulating Kapβ2 may mitigate neurotoxicity, a potential therapy for C9orf72-ALS/FTD.

## Introduction

The most common genetic cause of amyotrophic lateral sclerosis (ALS) and frontotemporal dementia (FTD) is a non-coding G_4_C_2_ nucleotide repeat expansion (NRE) in the first intron of the *C9ORF72* gene (C9)^1–3^. This NRE can be aberrantly translated through a process known as repeat-associated non-AUG translation (RAN-T), leading to the production of five different dipeptide repeat proteins (DPRs): poly glycine-alanine (GA), poly glycine-proline (GP), poly glycine-arginine (GR), poly proline-alanine (PA), and poly proline-arginine (PR)^4^. The arginine-rich DPRs, GR and PR, are the most neurotoxic, possibly in a length-dependent manner^5,6^. Studies in in-vitro models have identified dysregulated pathways associated with the accumulation of GR and PR, such as translation stalling, stress granules persistence, nucleolar stress, DNA damage, and nuclear pore complex defects^7–11^. A well-known pathological hallmark of most forms of ALS/FTD, including C9-ALS/FTD^12^, is the mislocalization of TDP-43 from the nucleus, where it usually resides, to the cytoplasm. Expression of long GR dipeptides (≥200) in cells causes TDP-43 nuclear depletion and accumulation of cytoplasmic TDP43 inclusions^12^. GR_80_-expressing mice show behavioral impairments but lack TDP-43 mislocalization in neurons^13^. Also, our recently established mouse model, which constitutively expresses GR_50_, develops mild motor behavioral phenotype^6^ but fails to display TDP-43 mislocalization in neurons. Interestingly, it showed disrupted nuclear pore complex integrity revealed in aged mice by Nup62 (nuclear pore complex protein) mislocalization in the cytoplasm^14^. From a mechanistic perspective, the influence of GR on nuclear import/export machinery remains unclear. Vanneste et al. showed that in-vitro expression of DPRs did not affect the nuclear import/export processes^15^, while Hayes et al. showed that R-rich DPRs disrupted the karyopherin-dependent nuclear import in a dose-dependent fashion^16^. Hutten et al., addressing specifically TDP-43 shuttling, showed that GR, even at a short length (GR_20_), hinders TDP-43 nuclear import^17^. In line with this evidence, it was demonstrated that GA can specifically cause TDP-43 nuclear import defects^18^. TDP-43 mislocalization is strictly a neuronal event, at least in the human disease context. However, these last two studies were conducted in human cancer cell lines (HeLa), whose protein and RNA composition greatly varies from neuronal cells.

The GR interactome is composed of many proteins, most of which feature low complexity domains, through which the interaction with GR occurs, including RNA-binding proteins (RBPs), nucleolar components, stress granules components, and proteins involved in nuclear transport^19,20^. Among the latter is karyopherin β2 (Kapβ2, also known as transportin-1 or Importin β2), which belongs to the family of nuclear import receptors (NIRs) that orchestrate the exchange of proteins between the nucleus and cytoplasm^21^. The flow direction depends upon the asymmetric distribution of RanGTP/RanGDP between the nucleus and cytoplasm. Kapβ2 translocates to the nuclear envelope^22,23^, where it can gain entry into the nucleus through interaction with the FG-Nups on the nuclear pore^24–27^. With this shuttling mechanism, Kapβ2 favors its entry into the nucleus when bound to its partners. Once in the nucleoplasm, Kapβ2 releases its cargo and relocates to the cytoplasm, freely transiting through the FG barrier of NPC channels while binding with the highly concentrated RanGTP. Once there, GTP is dephosphorylated in GDP, and Ran protein loses the interaction with Kapβ2, which is then ready for a new import cycle^21,25,28–30^.

Even though no ALS-causative mutations in the *KAPB2* gene have been identified thus far, the Kapβ2 protein interacts with many ALS-causative proteins such as FUS, but also TAF, EWS, hnRNPA1, and hnRNPA2^31–34^. Interestingly, the domain of these proteins involved in the interaction with Kapβ2 is a PY-NLS, a nuclear localization signal in which one proline (P) is followed by one tyrosine (Y), in which many of the most common and aggressive ALS-related mutations are found^35–37^. Even at very short lengths (e.g., 10 repeats), GR is predicted to interact with Kapβ2 *via* the same domain^16^. A recent study shows that PR also interacts with Kapβ2 through the sites that recognize the PY-NLS^38^. To add importance to the interaction of R-rich DPRs and Kapβ2, previous studies in yeasts showed that overexpression of KAP104, the yeast homolog of Kapβ2, decreased PR toxicity^39^, while in flies, Kapβ2 RNAi worsens PR neurotoxicity^19,40^.

In this work, we reported that GR interacts with Kapβ2 in primary neurons and CNS tissues, suggesting the relevance of this interaction to disease. We also explored the impact of this interaction on neuronal survival. Indeed, reduced levels of Kapβ2 had an adverse effect on the survival of neurons expressing GR. Conversely, increased Kapβ2 significantly mitigated GR neurotoxicity. These results indicate that Kapβ2 could be a modifier of a pathogenic disease mechanism. Therefore, manipulating its expression levels in neurons might be harnessed to design future therapies to improve their survival in C9orf72-linked neurodegeneration.

## Results

### Expression of GR does not alter endogenous levels of Kapβ2 in neurons

**Fig. 1:**
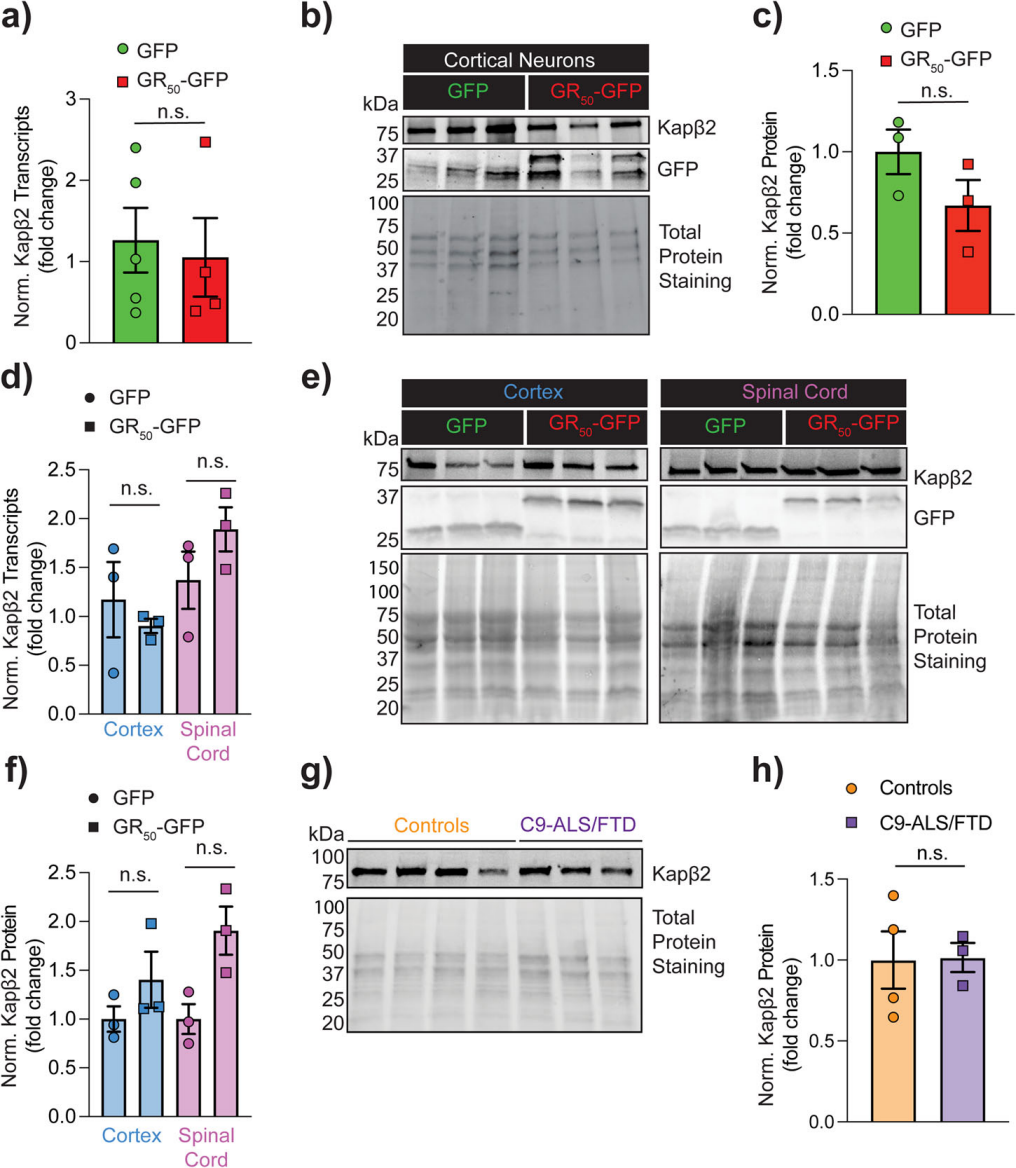
GR does not affect Kapβ2 levels in neurons. **a** qPCR analysis of Kapβ2 levels in cortical neurons transduced with GFP or GR_50_. Data are represented as mean ± S.E.M. (n = 6 biological replicates, Student *t*-test, n.s. not significant). **b** Homogenates of cortical neurons transduced with GFP or GR_50_ were resolved on western blot (WB) and probed for GFP and Kapβ2. Total protein staining was used as the loading control. For cortical neuron transduction experiments, we employed a lentivirus expression vector that encodes GR_50_ with Flag and GFP tags fused to the dipeptide N- and C-terminus, respectively. **c** The graph bar shows the quantification of the WB of cortical neuron extracts for Kapβ2 protein. Total protein staining was used to normalize. Data are represented as mean ± S.E.M. (n = 3 biological replicates, Student *t*-test, n.s. not significant). **d** qPCR analysis of Kapβ2 transcript levels in the cortex and spinal cord of GFP and GR_50_-GFP mice. Data are represented as mean ± S.E.M. (n = 3 biological replicates, One Way-ANOVA, n.s.) **e** Western blot of cortex and spinal cord homogenates from GFP and GR_50_ mice. GR_50_ is Flag and GFP tagged at the N- and C-termini, respectively. Blots were probed for GFP and Kapβ2. Total protein staining was used as the loading control. **f** The graph bar shows Kapβ2 protein quantification of the WB of cortex and spinal cord homogenates. Total protein staining was used as a loading control and normalizer. Data are represented as mean ± S.E.M. (n = 3 biological replicates, One Way-ANOVA, n.s.). **g** Western blot of controls and C9orf72 patient post-mortem cortex extracts. Total protein staining was used as the loading control. **h** Quantification of Kapβ2 expression levels in controls and C9orf72 patient post-mortem cortex extracts as normalized protein fold change. Data are represented as mean ± S.E.M. (n = 3 biological replicates, Student *t*-test, n.s. not significant).

Recruitment of essential constituents of the translational machinery by GR can lead to translation stalling, affecting the expression levels of certain proteins^41^. Thus, we first investigated whether the expression of GR could potentially affect Kapβ2 protein levels in our experimental models. For experiments in cortical neurons, we employed a lentivirus expression vector that encodes GR_50_ with Flag and GFP tags fused to the dipeptide N- and C-terminus, respectively, to aid with its visualization and identification when the encoding vector is transduced in cells. In addition, the lentivirus expression vector is driven by the neuronal-specific human synapsin promoter (hSyn). This promoter is active in neurons and will only drive gene expression in these cell types. In cortical neurons transduced with GR_50_, there was no significant change in Kapβ2 mRNA compared to neurons expressing GFP used as reference (Fig. 1a, b; Supplementary Fig. 8). When we measured Kapβ2, we observed a trend in reduction in Kapβ2 protein levels in samples transduced with GR_50_ compared to GFP alone, which did not reach statistical significance (Fig. 1c).

Moreover, we assessed Kapβ2 mRNA and protein levels in the cerebral cortex and spinal cord homogenates obtained from a transgenic mouse expressing GR_50_ or GFP^6^. This analysis revealed no significant differences between the two groups in Kapβ2 mRNA and protein levels (Fig. 1d–f; Supplementary Fig. 8). We also assessed if there were discrepancies in the expression levels of the GR_50_ and GFP transcripts. Still, we did not find significant changes between the experimental groups in cortical neurons and mouse CNS tissues (Supplementary Fig. 1a, b). While post-mortem tissues of C9orf72-ALS/FTD patients contain GR-positive aggregates, whose abundance varies in different brain regions^42^, we found that Kapβ2 protein expression levels were not changing, as determined by a western blot analysis of homogenates obtained from the motor cortex of C9orf72-ALS/FTD and non-diseased control cases (Fig. 1g, h; Supplementary Fig. 8). We also analyzed the expression of the Kapβ2 transcripts in publicly available datasets, including the one from the *Answer ALS consortium* (transcriptomic data from iPS-derived cortical neurons (iCN) from ALS patients) and the ALS Cell Atlas (frontal cortex and cerebellum transcriptomic data from ALS patients and controls). We found no differences in Kapβ2 RNA transcript levels when comparing control and C9orf72-ALS/FTD groups (Supplementary Fig. 8c, d).

### GR interacts with Kapβ2 in neurons

**Fig. 2:**
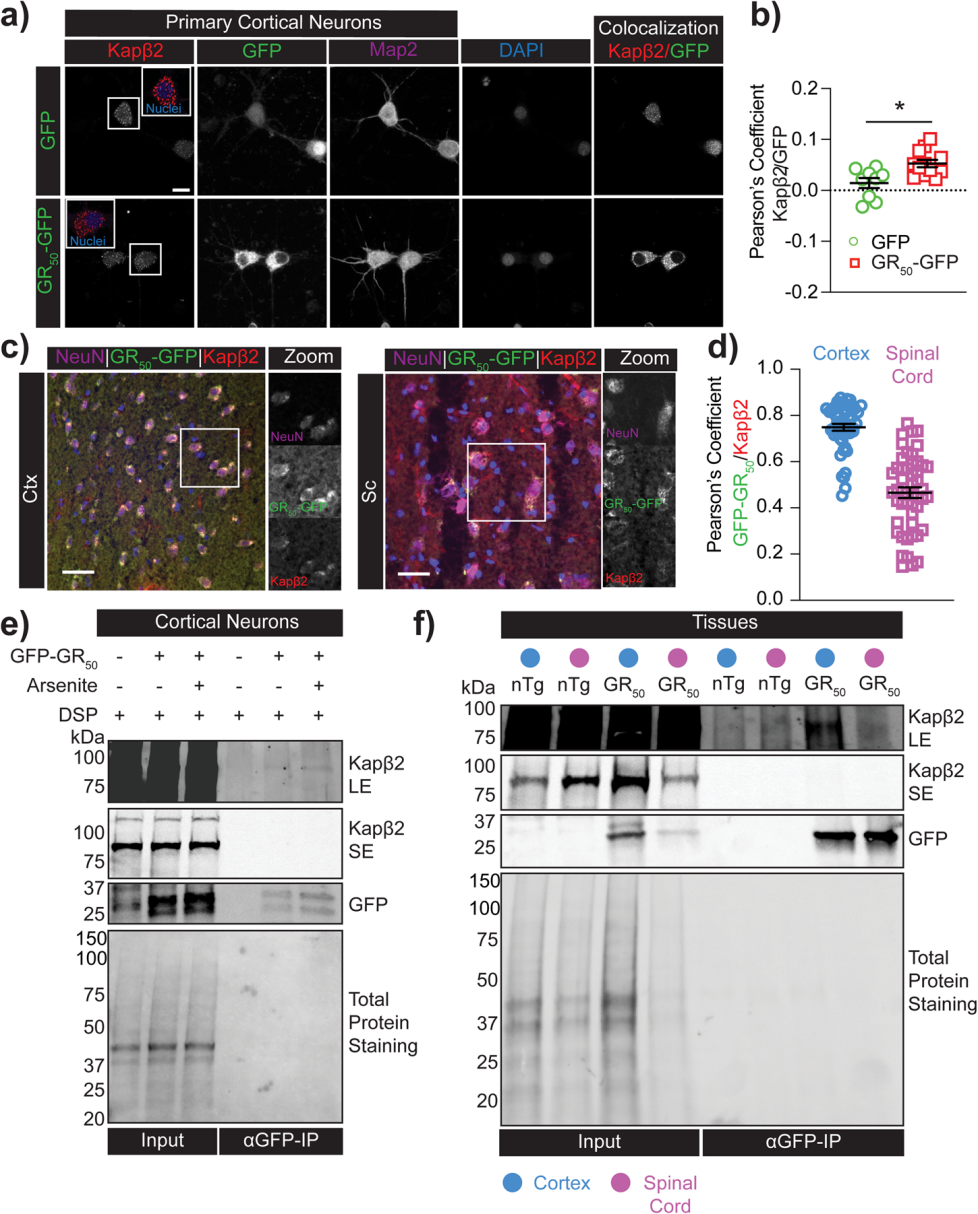
GR interacts and recruits Kapβ2 in neurons. **a** Confocal images of rat cortical neurons in culture transduced with GFP and GR_50_ and stained for Kapβ2 and Map2. Imaris colocalization channel shows the amount of GFP colocalized with Kapβ2. Scale bar = 10 μm. **b** Pearson’s coefficient measured in each neuron transduced with the lentivirus GR_50_ construct (Flag-GR_50_-GFP). Data are represented as mean ± S.E.M. (n = 3 biological replicates, m > 5 neurons, Student *t*-test, *P < 0.05). **c** Confocal images of the cortex and spinal cord tissues of GR_50_ mice stained for Kapβ2. Green is GFP reporting for GR_50_, red is Kapβ2, and magenta is NeuN. Imaris colocalization shows the amount of GFP colocalized with Kapβ2. Scale bar = 30 μm. **d** Pearson’s coefficient measured in the cortex and spinal cord neurons of GR_50_ mice. Data are represented as mean ± S.E.M. (n = 3 biological replicates, m > 15 neurons). **e** Western blot analysis of the immunoprecipitation assay was performed in rat cortical neurons transduced with GR_50_ and treated with arsenite (0.5 mM; 30 min). Blots were probed for GFP and Kapβ2. Total protein staining was used as the loading control. SE short exposure time, LE long exposure time. **f** Western blot of the immunoprecipitation assay performed in the cortex or spinal cord tissue of nTg or GR_50_ mice. Blots were probed for GFP and Kapβ2. Total protein staining was used as the loading control. SE short exposure time, LE long exposure time.

Earlier investigations utilizing pull-down assays revealed that Kapβ2 and GR interacts in various human cell line models^16,17^. To explore whether this interaction would also occur in cell types more relevant to the disease, we employed a lentivirus expression vector to transduce primary cortical neurons with GR_50_ (Flag and GFP tagged). Immunofluorescence staining for Kapβ2 showed its perinuclear and cytoplasmic localization as expected (Fig. 2a). We also observed that GR_50_ localization was predominantly cytoplasmic or perinuclear, similar to what we previously reported for our knock-in GR_50_ (with Flag and GFP tags) mice^6^, where the expression of transgene is approximately at physiological levels. We then measured the Pearson’s correlation coefficient in the cytoplasm of transduced neurons to evaluate whether GR_50_ and Kapβ2 co-localize and estimated the degree of co-localization of these proteins in the same cellular compartments. Although the measured Pearson’s coefficient yielded a low score, which verges on potential biological negligence according to the field^43^, it’s important to keep in mind that further analysis and interpretation may be required to determine its significance. Our analysis has shown that the co-localization coefficient was higher in GR_50_ neurons in comparison to neurons that were transduced with GFP. This indicates a stronger correlation between GR_50_ and Kapβ2, as shown in Fig. 2b, than with GFP alone. Therefore, we interpret the low Pearson’s coefficient score for the interaction of GR_50_ with Kapβ2 as a reflection of the dynamic interaction between these two proteins. We measured the fluorescence of Kapβ2 in the nucleus and in the cytoplasm of neurons transduced with GR_50_ and GFP alone. We observed a slight but non-significant decrease of Kapβ2 fluorescence in GR_50_ compared to GFP samples, paralleled by a significant increase in cytoplasmic Kapβ2 fluorescence (Supplementary Fig. 2a). Plotting the nucleo-cytoplasmic ratio, we observed a substantial decrease in the GR_50_ samples. This data suggests GR interferes with Kapβ2 localization in neurons, favoring its cytoplasmic localization.

Next, to investigate whether Kapβ2/GR also interacted in in-vivo models, we used a recently published GR_50_ transgenic mouse model we generated and characterized^6^. This mouse model expresses Flag-GR_50_-GFP predominantly in neurons. We first assessed the Kapβ2 localization pattern in wild-type, non-transgenic mice. We found that Kapβ2 mainly localized in perinuclear regions and cytoplasm of CNS cells (Supplementary Fig. 2b). We then performed Kapβ2 immunofluorescence staining in cerebral cortex and spinal cord sections of the GR_50_ transgenic mouse employing Imaris software to perform 3D rendering and visualize the Kapβ2 localization in the cortex and spinal cord neurons. We found robust co-localization of Kapβ2 and GR_50_ (Pearson’s coefficient ranging between 0.5 and 1 in both tissues) in NeuN-positive cells (Fig. 2c, d; Supplementary Fig. 2c; Supplementary Movie 1). We also observed a higher Pearson’s coefficient in cortical neurons than in motor neurons, possibly due to the different expression levels of Kapβ2 in the two cell types (https://www.proteinatlas.org/ENSG00000083312-TNPO1).

Further confirmation for GR_50_ and Kapβ2 reciprocal interaction in neurons and CNS tissues came from co-IP experiments. However, this assay posed a challenge, as the fluorescent GFP reporter interacts with numerous proteins in cells. A dataset containing over 2900 protein entries found to interact with both GFP and GR_50_ was recently published^44^ (MassIVE identifier MSV000088581) (Supplementary Fig. 3a). We plotted the intensities of the interacting proteins and found that Kapβ2 and Kapβ2b were significantly enriched in the GR_50_ group compared to the GFP group (Supplementary Fig. 3b), suggesting that Kapβ2 and GR_50_ could be interacting partners. To further study this interaction, we carried out co-IP in GR_50_ transduced cortical neurons. Before harvesting the neurons, we treated the sample with the crosslinker DSP to preserve even the most dynamic and transient interactions between these two proteins. In immunoprecipitates from neurons expressing GR_50_, we observed Kapβ2. Its interaction with GR_50_ increased in neurons treated with the cellular stressor, arsenite (Fig. 2e; Supplementary Fig. 8). We performed the same co-IP in control GFP-expressing neurons. As expected, a fraction of Kapβ2 was non-specifically bound to GFP, but a larger fraction was bound to GR_50_ (Supplementary Fig. 3c, d). Kapβ2 and GR interaction was also confirmed by co-IP using anti-FLAG M2 agarose beads to pull down GR_50_ (Supplementary Figs. 4a, 8). Finally, we conducted reverse co-IP experiments by using a Kapβ2 antibody to capture GR. Our results showed that a portion of GR was co-immunoprecipitated with Kapβ2, especially in arsenite-stressed neurons (Supplementary Figs. 4b, 8).

Co-IP experiments were also conducted in cortex and spinal cord lysates obtained from transgenic Flag-GR_50_-GFP mice^6^. As seen in cultured neurons, Kapβ2 co-immunoprecipitated with GR_50_ when we used an anti-GFP antibody but not in non-transgenic control CNS tissue lysates or when we used for co-IP the corresponding IgG control (Fig. 2f and Supplementary Figs. 4c, 8). In addition, we performed a reverse co-IP using a Kapβ2 antibody, which also revealed evidence of GR bound to Kapβ2 (Supplementary Figs. 4d, 8).

### Kapβ2 is protective against GR-mediated neurotoxicity

**Fig. 3:**
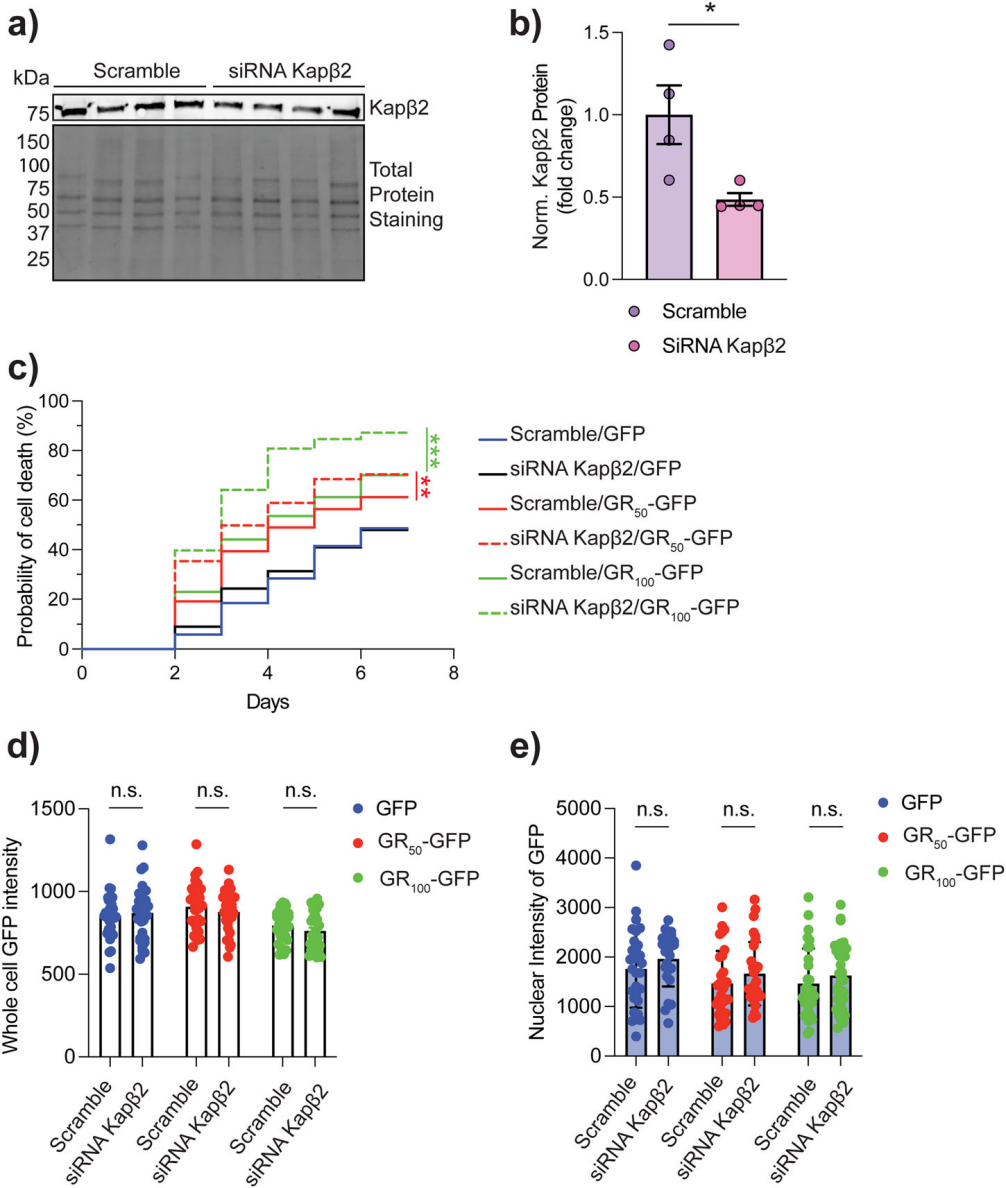
Silencing of Kapβ2 increases death risk in the presence of GR. **a** Western blot showing Kapβ2 expression in neurons treated with Smart pool siRNA (100 μM). Blots were probed for Kapβ2. Total protein staining was used as the loading control. **b** Kapβ2 levels in primary neurons treated with Smart pool siRNA 100 μM or scramble. Data are represented as mean ± S.E.M. (n = 3 biological replicates, Mann-Whitney non parametric test, *P* < 0.05). **c** The plot depicts the probability of neuronal death in each group via a cumulative risk of death plot. Time-lapse experiments on rat primary cortical neurons transfected with siRNA Kapβ2 100 μM or scramble and GFP, GR_50_-GFP, or GR_100_-GFP. (n = 3 biological replicates, m > 50 neurons, Log-rank Mantel-Cox test *p < 0.05, ***p < 0.001). **d** Quantification of the mean fluorescence of GFP expressing neurons 48 h after transfection in cells previously treated with Kapβ2 siRNA or scramble. Data are represented as mean ± S.E.M. (n = 3 biological replicates, m > 10 neurons, One-Way ANOVA, *p* = n.s. not significant). **e** Quantification of the mean nuclear fluorescence of GFP expressing cells 48 h after transfection in cells previously treated with Kapβ2 siRNA or scramble. Data are represented as mean ± S.E.M. (n = 3 biological replicates, m > 10 neurons, One-Way ANOVA, *p* = n.s. not significant).

Next, we conducted a study to investigate if the Kapβ2-GR interaction affects the toxicity of GR in neurons. Our goal was to diminish the expression of Kapβ2 in neurons that express GR and document its impact on their survival. To achieve this, we used a combination of 4 siRNAs that specifically target Kapβ2 RNA transcripts. The transfection of these siRNAs resulted in a substantial decrease of Kapβ2 protein levels by approximately 50% after 48 h (Fig. 3a, b; Supplementary Fig. 8). Using live-cell imaging microscopy, we measured a significant reduction in viability in the GR_50_ and GR_100_ (GFP tagged) expressing neurons in which Kapβ2 was silenced (Fig. 3c, Supplementary Fig. 5c, Supplementary Table 1). We measured the fluorescence intensity of GFP in all experimental groups 48 h after transfection to determine if Kapβ2 knockdown impacted the expression levels of GR_50_ and GR_100_. No difference was observed (Fig. 3d) even when we looked at the nuclear accumulation of GR_50_ and GR_100_ (Fig. 3e), suggesting that Kapβ2 is not involved in GR degradation or subcellular localization. Similarly, knocking down Kapβ2 in neurons did not affect nuclear FUS levels, a known and extensively studied substrate for Kapβ2 and an ALS-causative protein when mutated^34^, measured by quantifying the FUS immunofluorescence signal (Supplementary Fig. 8a, b). These results led us to hypothesize that increasing the levels of Kapβ2 might play a protective role against GR-mediated neurotoxicity.

**Fig. 4:**
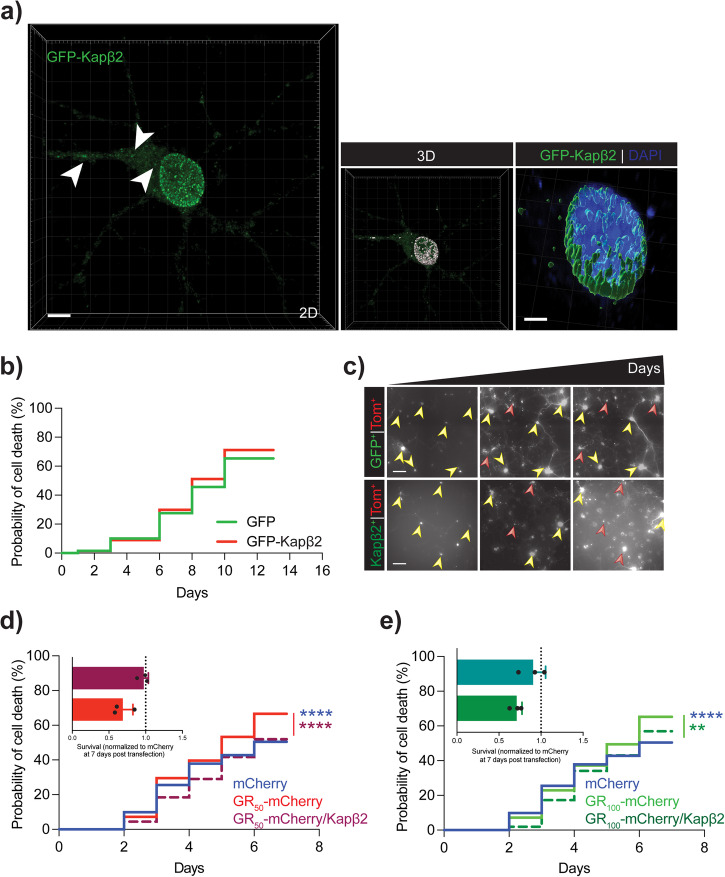
Increased expression of Kapβ2 does not affect neuronal viability. **a** Left: Confocal imaging of primary neurons transfected with GFP-Kapβ2. Arrows indicate cytoplasmic puncta. Green is GFP, and blue is DAPI. Scale bar = 5 μm. Center: Imaris 3D rendering of GFP-Kapβ2 expression in primary rat cortical neurons. Right: Magnification of Imaris’ rendering of GFP-Kapβ2 puncta around the nucleus. Scale bar = 3 μm. **b** Probability of neuronal death via a cumulative risk of death plot of rat primary cortical neurons transfected with 200/400 ng of Tm^+^/ GFP-Kapβ2^+^ (GFP used as control) per 150,000 cells. Neurons double positive Tm^+^/GFP^+^ were counted (n = 3 biological replicates, m > 150 neurons, Log-rank Mantel-Cox test: n.s. not significant). **c** Representative images of neurons over time transfected with 200/400 ng of Tm^+^/GFP^+^. Green is Kapβ2; red is Td-Tomato. Scale bar = 20 μm. **d** Probability of neuronal death via a cumulative risk of death plot of rat primary cortical neurons transfected with 400/400 ng of GFP-Kapβ2 (GFP alone used as control) and GR_50_-mCherry (mCherry alone used as control) per 150,000 cells. Neurons double positive GFP^+^/mCherry^+^ were counted (n = 3 biological replicates, m > 150 neurons, Log-rank Mantel-Cox test: mCherry vs GR_50_-mCherry *p* < 0.0001; GR_50_-mCherry vs GR_50_-mCherry /GFP-Kapβ2 *p* < 0.0001). Inset: bar graph showing quantification of GR_50_-mCherry and GR_50_-mCherry /GFP-Kapβ2 overall survival normalized to mCherry 7 days post-transfection, n = 3 biological replicates. Data are represented as mean ± S.E.M. **e** Probability of neuronal death via a cumulative risk of death plot of rat primary cortical neurons transfected with 400/400 ng of GFP-Kapβ2^+^ (GFP alone used as control) and GR_100_-mCherry (mCherry alone used as control) per 150,000 cells. Neurons double positive GFP^+^/mCherry^+^ were counted (n = 3 biological replicates, m > 150 neurons, Log-rank Mantel-Cox test: mCherry vs GR_100_-mCherry *p* < 0.0001; GR_100_-mCherry vs GR_100_-mCherry/Kapβ2 *p* = 0.005). Inset: bar graph showing quantification of GR_100_-mCherry and GR_100_-mCherry /GFP-Kapβ2 overall survival normalized to mCherry at 7 days post-transfection, n = 3 biological replicates. Data are represented as mean ± S.E.M.

Next, we co-expressed GFP-tagged Kapβ2 and GR_50__-100_-mCherry in primary cortical neurons to test the hypothesis that Kapβ2 plays a neuroprotective role and assessed the viability of the co-transfected neurons in culture. Heterologously expressed Kapβ2 localized in cytoplasmic puncta that mainly surrounded the nucleus of neurons (Fig. 4a, Supplementary Movie 2). We first determined that Kapβ2 or GFP alone were not toxic to neurons when heterologously expressed at two different DNA concentrations (400 and 800 ng/well) (Fig. 4b, c; Supplementary Fig. 6a, b; Supplementary Table 2). This suggested that elevating Kapβ2 expression does not adversely affect neuronal viability. Next, we expressed both Kapβ2 and GR_50_ or GR_100_ and assessed the overall risk of death of the transfected neurons. We kept a 1:1 DNA ratio of Kapβ2-GFP (or GFP alone as control) and GR-mCherry (or mCherry alone), and GFP/mCherry-positive neurons were imaged and analyzed over 8 days (Fig. 4d, e; Supplementary Fig. 6c; Supplementary Table 3). Expression of GR_50_ or GR_100_ in neurons increased their risk of death compared to their respective controls (mCherry vs. GR_50_-mCherry *p* < 0.0001; mCherry vs. GR_100_-mCherry *p* < 0.0001) as previously reported^5,6^. However, co-expression of Kapβ2 in GR_50-100_ (mCherry tagged) neurons reduced their overall risk of death to control levels (GR_50_-mCherry vs. GR_50_-mCherry/Kapβ2 *p* < 0.0001; GR_100_-mCherry vs. GR_100_-mCherry/Kapβ2 *p* = 0.005) (Fig. 4d, e; Supplementary Fig. 6c; Supplementary Table 3) indicating that Kapβ2 can indeed rescue neurons from the intrinsic toxicity of GR expression.

### Kapβ2 exhibits localization to GR aggregates but doesn’t affect GR aggregation propensity, GR aggregates dynamic properties, and GR localization pattern in neurons

**Fig. 5:**
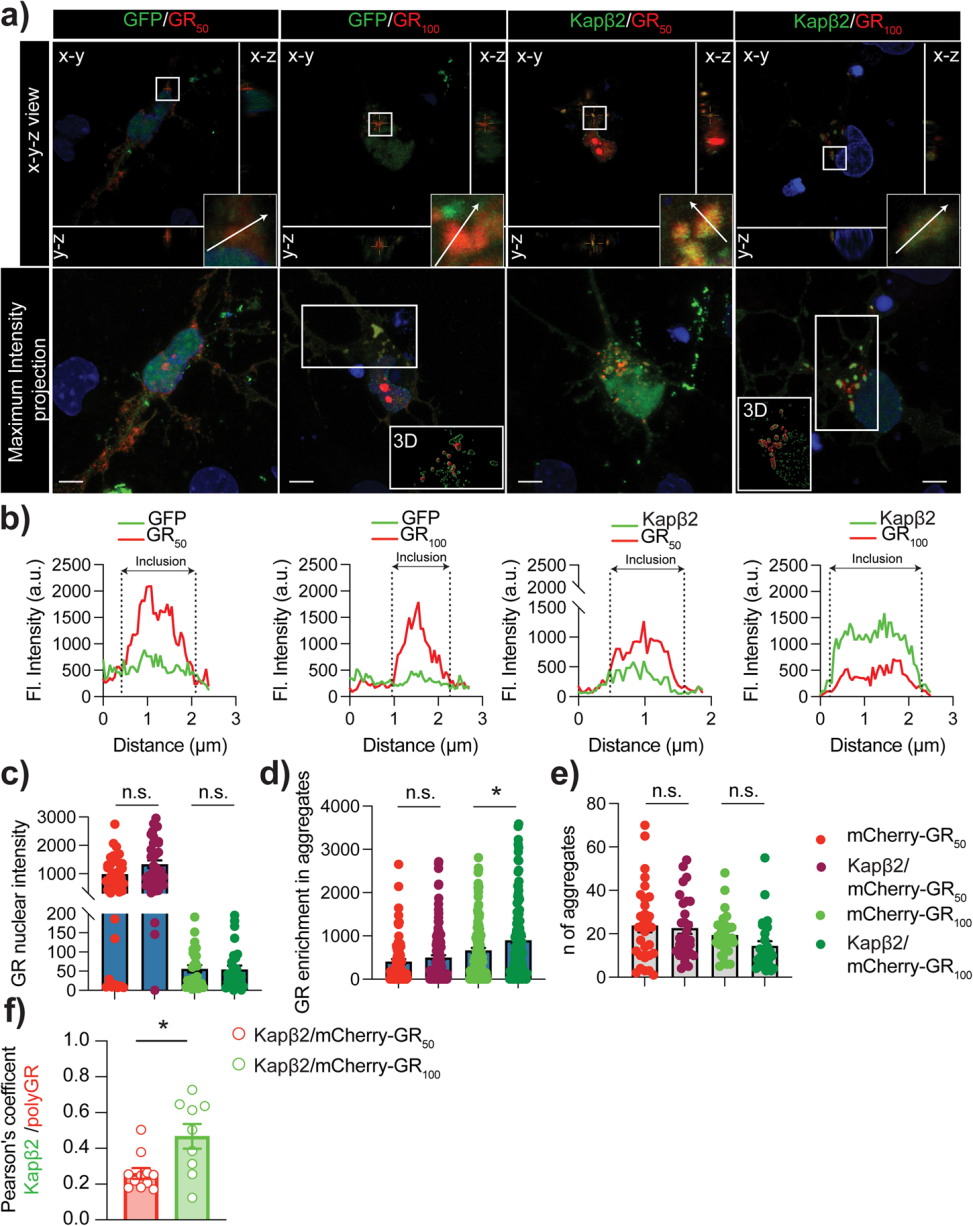
Increased expression of Kapβ2 mitigates GR neuronal toxicity. **a** Confocal analysis of neurons overexpressing GFP or Kapβ2 and GR_50_-mCherry or GR_100_-mCherry. The top row shows an x-y-z view, while the bottom row shows the maximum intensity projection of the Z plans acquired. Green is GFP, red is mCherry, and blue is DAPI. The white line represents the length over which GFP and mCherry fluorescence were analyzed. Scale bar = 10 μm. **b** Graph showing intensity profiles of GFP or mCherry fluorescence (a.u.) over distance (μm). **c** The bar graph shows quantification of GR_50_-mCherry and GR_100_-mCherry nuclear intensity Data are represented as mean ± S.E.M. Only double positive cells were considered. (n = 3 biological replicates, m = at least 30 neurons/group, One-Way ANOVA, Tukey’s multiple comparisons test, n.s. not significant). **d** The bar graph shows the quantification of GR_50_-mCherry and GR_100_-mCherry mean fluorescence intensity in GR-positive granules. Data are represented as mean ± S.E.M. Only double positive cells were considered. (n = 3 biological replicates, m = at least 30/group neurons, One-Way ANOVA, Tukey’s multiple comparisons test, n.s. not significant). **e** The bar graph shows the quantification of the GR_50_-mCherry and GR_100_-mCherry aggregates per cell. Data are represented as mean ± S.E.M. Only double positive cells were considered. (n = 3 biological replicates, m = at least 30 neurons/group, One-Way ANOVA, Tukey’s multiple comparisons test, n.s. not significant). **f** Pearson’s coefficient analysis of GFP-Kapβ2 and GR_50_-mCherry or GR_100_-mCherry. Data are represented as mean ± S.E.M. (n = 3 biological replicates, m = 9–10 neurons/group, Student *t*-test, *P* < 0.05).

To explore the mechanism by which Kapβ2 protects neurons from GR toxicity, we first evaluated whether Kapβ2 affected the formation and stability of GR aggregates. In neurons, the subcellular localization of GR_50_ and GR_100_ differs significantly. GR_50_ is predominantly located in the nucleus, while GR_100_ is mainly in the cytoplasm (Fig. 5a and Supplementary Fig. 6d). Despite both GR species form aggregates in the cytoplasm, with no observable difference in the number of aggregates per cell between GR_50_ and GR_100_ expressing neurons (Fig. 5e), we analyzed the intensity profile of GFP (reporting on the transfected Kapβ2) and mCherry (reporting on the transfected GRs) channels in the GR aggregates. We observed selective localization of Kapβ2 within aggregates, as opposed to cytoplasmic diffusion like GFP, as shown in Fig. 5a, b. Furthermore, the fluorescence intensity profile of the aggregates indicated that the recruitment of Kapβ2 was even higher in GR_100_-expressing neurons, suggesting that Kapβ2 could ultimately affect GR localization. However, we found no difference in GR fluorescence within the nuclei in the presence of Kapβ2, as illustrated in Fig. 5c. Additionally, we found that the mean fluorescence of each aggregate remained constant when Kapβ2 was recruited into them, as shown in Fig. 5d. Kapβ2 did not alter the number of aggregates per cell for either GR species (Fig. 5e). Finally, by calculating the Pearson’s correlation coefficient to assess whether the recruitment and colocalization of Kapβ2 and GR depended on the repeat length, we found that GR_100_ aggregates displayed a more substantial positive correlation value than GR_50_ (Fig. 5f).

**Fig. 6:**
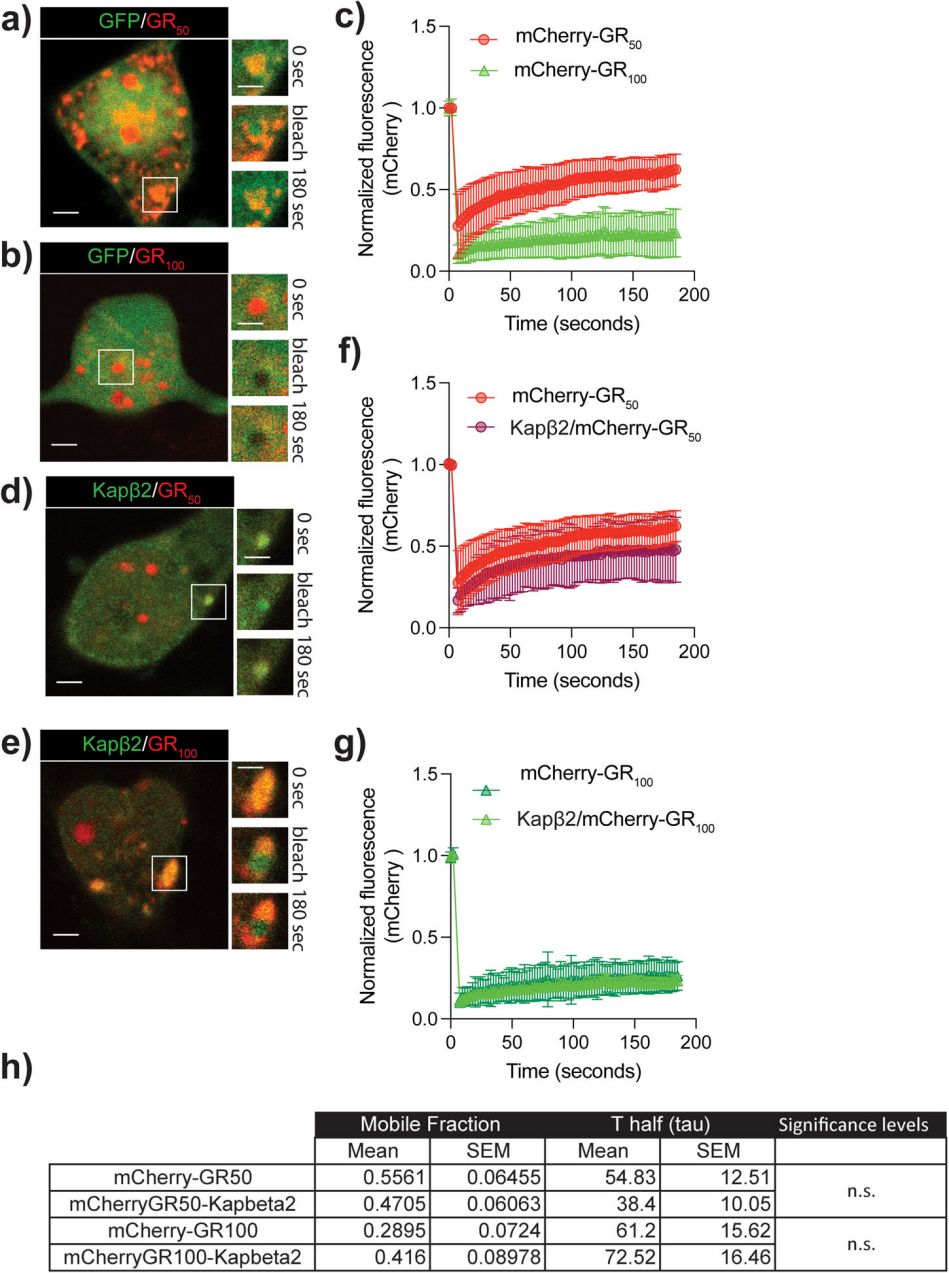
Kapβ2 does not change the dynamic properties of GR aggregates. **a** Representative images of the GR_50_-mCherry bleached aggregates at the start (0 s), bleaching (5 s), and end (180 s). Scale bar = 10 μm. **b** Representative images of the GR_100_-mCherry bleached aggregates at the start (0 s), bleaching (5 s), and end (180 s). Scale bar = 10 μm. **c** The graph shows fluorescence recovery over time for GR_50_-mCherry and GR_100_-mCherry. Data are represented as mean ± S.E.M. (n = 3 biological replicates, m > 5 neurons, Student *t*-test, *P* < 0.001). **d** Representative images of the GFP-Kapβ2/GR_50_-mCherry bleached aggregates at the start (0 s), bleaching (5 s), and end (180 s). Scale bar = 10 μm. **e** Representative images of the GFP-Kapβ2/ GR_100_-mCherry bleached aggregates at the start (0 s), bleaching (5 s), and end (180 s). Scale bar = 10 μm. **f** The graph shows fluorescence recovery over time for GR_50_-mCherry and GFP-Kapβ2/GR_50_-mCherry. Data are represented as mean ± S.E.M. (n = 3 biological replicates, m > 5 neurons). **g** The graph shows fluorescence recovery over time for GR_100_-mCherry and GFP-Kapβ2/GR_100_-mCherry. Data are represented as mean ± S.E.M. (n = 3 biological replicates, m > 5 neurons). **h** A table summarizing mean and S.E.M. of the mobile fraction, t1/2 (tau), and the significance level for the FRAP curve profiles presented in (**f**) and (**g**); n.s. not significant.

Kapβ2 affects the dynamic properties of FUS aggregates^37^, as demonstrated by its ability to modulate their physical properties, liquid phase separation, and formation of insoluble gel-like structures^34^. To investigate whether Kapβ2 similarly influences the GR aggregates, we co-expressed Kapβ2-GFP and GR_50_ or GR_100_-mCherry in cortical neurons and used fluorescence recovery after photobleaching (FRAP) to assess the overall fluidity of the aggregates (Fig. 6a–c; Supplementary Fig. 6e). We observed that the mCherry fluorescence recovery was significantly higher in GR_50_-mCherry when compared to GR_100_-mCherry aggregates. This observation led us to conclude that the aggregates formed by GR_100_-mCherry were more stable (Fig. 6a–c). Notably, in the presence of Kapβ2-GFP, the fraction of mCherry fluorescence recovery remains unchanged for both GR_50_-mCherry and GR_100_-mCherry aggregates, suggesting that Kapβ2 does not bring the aggregates in a more dynamic and fluid state (Fig. 6d–h; Supplementary Fig. 6e). These findings indicate that Kapβ2 when co-aggregates with GR, does not alter the structure of the inclusions, confirming what shown in silico by ref. ^17^.

**Fig. 7:**
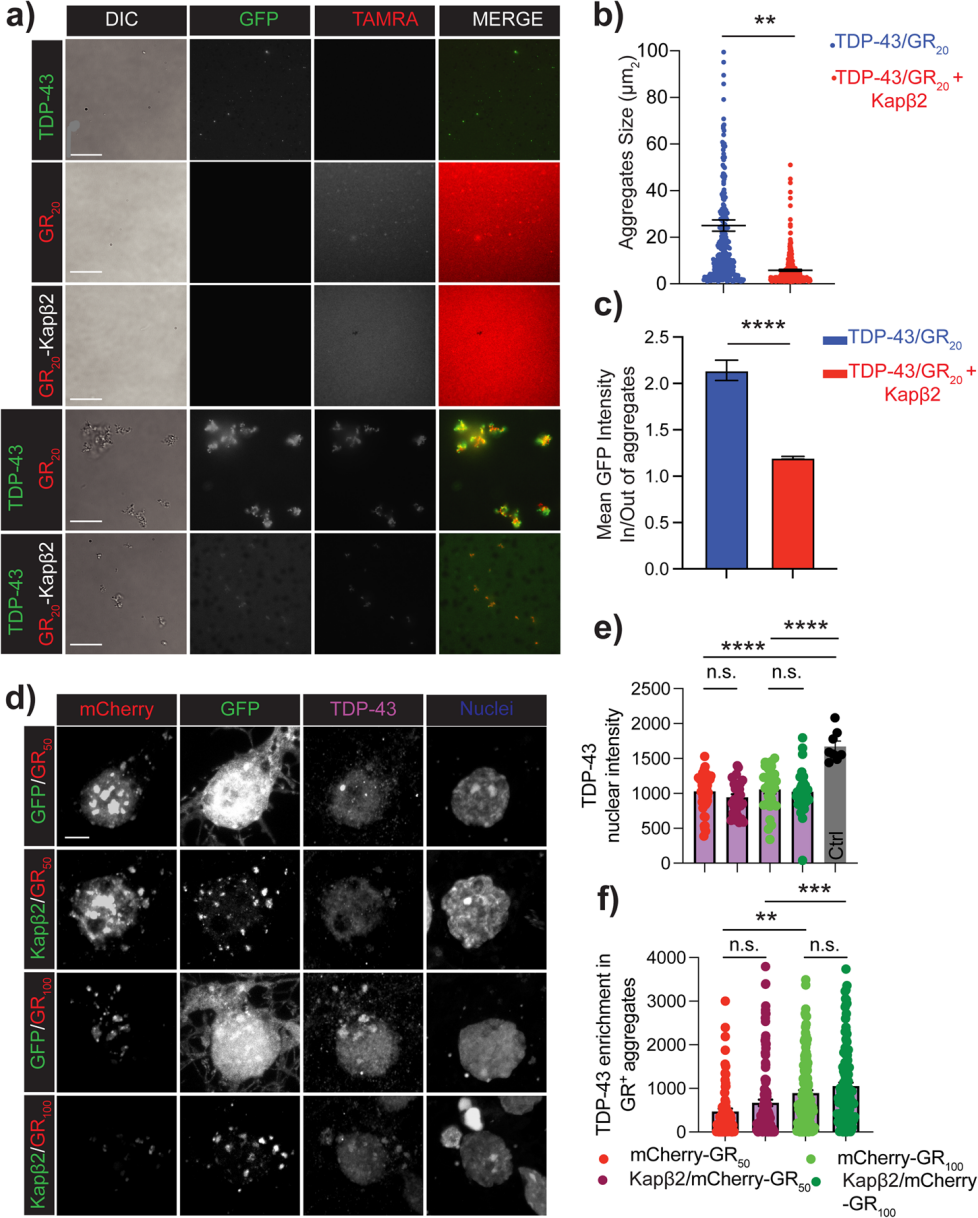
Increasing Kapβ2 expression levels does not affect TDP-43 subcellular localization in the presence of GR. **a** Epifluorescence images of condensate formation in vitro in a test tube containing equimolar concentration (5 μM) ofTDP-43, GR_20,_ and Kapβ2. Green is TDP-43, and red is GR_20_. Scale bar = 15 μm. **b** Quantification of the size of TDP-43/GR_20_ aggregates in the presence or absence of Kapβ2. Data are represented as mean ± S.E.M. (n = 3 biological replicates, Student *t*-test, *P* < 0.01). **c** Ratio between the mean GFP intensity inside and outside TDP-43/GR_20_ aggregates in the presence or absence of Kapβ2. Data are represented as mean ± S.E.M. (n = 3 biological replicates, Student *t*-test, *P* < 0.0001). **d** Confocal analysis of neurons overexpressing GFP or Kapβ2 and GR_50_-mCherry (top row) or GR_100_-mCherry (bottom row). Scale bar = 5 μm. **e** Single-nuclei TDP-43 staining quantification. Data are represented as mean ± S.E.M. Only double positive cells were considered. (n = 3 biological replicates, m = at least 9 neurons/group, Student *t*-test, *P* < 0.01; *P* < 0.001). **f** TDP-43 quantification into GR aggregates. Data are represented as mean ± S.E.M. Only double positive cells were considered. (n = 3 biological replicates, m = at least 30 neurons/group, Student *t*-test, *P* < 0.01; *P* < 0.001).

In the presence of GR, Kapβ2 is crucial in regulating TDP-43 localization, although it does not directly bind to TDP-43^17^. We conducted experiments in vitro, in a test tube, where GR/TDP-43 aggregates were allowed to form in the presence and absence of Kapβ2. We used an equimolar concentration (5 μM) for all three proteins. When combined, GR and TDP-43 formed large condensates (Fig. 7a, Supplementary Fig. 7). It is interesting to note that the introduction of Kapβ2, but not an unrelated protein like BSA (5 μM), reduced the size of the aggregates, enriched TDP-43 within the aggregates, and increased the diffusive TDP-43 fluorescence signal outside the aggregates (as shown in Fig. 7b, c). This suggests that Kapβ2 made a portion of TDP-43 soluble. We then hypothesized that the fraction of TDP-43 freed from the aggregates by Kapβ2 would be available to re-enter the nucleus freely. This hypothesis was tested in primary rat neuronal cultures. We measured intranuclear TDP-43 in neurons co-transfected with GR_50,100_-mCherry and Kapβ2-GFP or GFP as control and found that TDP-43 was significantly depleted from the nucleus of neurons expressing GR_50,100_ compared to GFP-expressing control neurons (Fig. 7d, e). Under this experimental condition, the expression of Kapβ2-GFP did not revert TDP-43 nuclear loss caused by the presence of GR. We thus analyzed TDP-43 content in GR aggregates, and as expected, we found that GR aggregates were enriched in TDP-43. However, we also found that the fraction of TDP-43 in the aggregates did not change in the presence of Kapβ2 (Fig. 7d–f). Overall, while in neurons Kapβ2 makes GR aggregates more dynamic, it does not influence TDP-43 relocalization to the nucleus or the aggregates.

## Discussion

Our study demonstrated the impact of modifying Kapβ2 levels on GR-mediated neuronal toxicity. We established that GR interacts with Kapβ2 in-vitro in primary neuronal culture and in-vivo in CNS tissues, such as the cortex and spinal cord of a GR-expressing transgenic knock-in mouse model. These studies were facilitated by the fusion of the GR protein to the GFP molecule, and thus, we have provided GFP-only controls for all the experiments to show the specificity of our findings for GR. Interaction studies in vivo are presented in this paper always using orthogonal methods aimed at making the claims more robust. For example, in animal tissues, the co-IP data was analyzed by western blot, which supported the colocalization data analyzed by confocal microscopy. We didn’t image the tissue of GFP-only mice using confocal microscopy due to the inefficient fluorescence signal of the GFP reporter^6,14^.

Kapβ2 and GR have been implicated in the pathogenesis of certain forms of ALS and FTD. Kapβ2 is a nuclear import receptor that regulates the transport of substrate molecules critical for cell survival between the nucleus and the cytoplasm^35^. Dysregulation of nuclear-cytoplasmic transport is thought to contribute to the pathogenesis of ALS and FTD, and it is possible that an interaction between Kapβ2 and GR could have implications for this process. Indeed, we found that suppressing Kapβ2 levels elevates the risk of neuronal death. On the other hand, Kapβ2 overexpression shields neurons against GR-induced neurodegeneration.

The formation of GR aggregates is believed to be linked to several cellular dysfunctions in C9orf72-ALS/FTD, including impaired nucleo-cytoplasmic transport, altered proteostasis and RNA metabolism. Dysregulation of these cellular processes can lead to the accumulation of toxic protein inclusions, including GR aggregates, which can trigger a cascade of toxic events that lead to neuronal dysfunction and death. We demonstrated that Kapβ2 exhibits enrichment within GR aggregates but that its presence does not modify the mobile fraction of GR within the aggregates. To resolve protein aggregates, it’s often necessary to increase their mobility. This allows other proteins, which are responsible for clearing the inclusion, to access the aggregates and extract the proteins that need to be degraded. This can facilitate the disassembly and degradation of the aggregates, freeing co-aggregated proteins essential for cell survival, such as TDP-43^45^. We were thus not surprised to observe that Kapβ2 did not alter TDP-43 localization in the GR aggregates. TDP-43 mislocalization is a pathological feature of the majority of ALS cases. It refers to the abnormal accumulation or redistribution of TDP-43 in cytoplasmic inclusions in affected neurons and its depletion from the nucleus, where it physiologically resides^46^. TDP-43 mislocalization triggers a cascade of toxic events that lead to neuronal dysfunction and death^47,48^. Hence, potential therapeutic strategies for ALS could be directed to prevent TDP-43 mislocalization and/or restore its nuclear localization. Several studies, including the present one, have also demonstrated a physical interaction between GR and TDP-43. For example, a study by ref. ^10^ showed that GR directly interacted with TDP-43 and sequestered it in cytoplasmic aggregates, leading to its depletion from the nucleus. This mislocalization of TDP-43 is thought to be a critical step in the pathogenesis of ALS and FTD.

In our in-vitro assay, we found that the addition of Kapβ2 hindered the entry of TDP-43 into the GR aggregates and allowed TDP-43 to remain soluble in the buffer. However, we failed to validate this mechanism in primary neurons in culture. Indeed, we did not observe an increase in nuclear TDP-43 re-localization. Interestingly, these results contradict the previous findings by Hutten et al., which showed that Kapβ2 could directly disrupt GR/TDP-43 co-aggregation, restoring TDP-43 nuclear localization. The ability of DPRs to disrupt the nucleo-cytoplasmic transport and indirectly promote TDP-43 aggregation was also shown by a recent study in HeLa cells^18,49^. In our neuronal culture model, we observed co-aggregation of TDP-43 with GR_50_ and GR_100_ and a corresponding decrease in nuclear TDP-43. While Kapβ2 also localizes to the GR aggregates, it does not reduce TDP-43 localization in the aggregates or alter the TDP-43 nucleo/cytoplasmic ratio.

Numerous studies in the ALS/FTD field have focused on the disruption of the nuclear pore complex and the nuclear import/export activity^7,50,51^. Defects in the proper import/export of critical proteins are associated with DPRs or G_4_C_2_ repeat-containing C9orf72 RNA transcripts. At the same time, TDP-43 aggregates trigger defects in nucleo-cytoplasmic transport, highlighting the importance of these pathways in disease^16,52^. Therefore, conducting studies that further characterize these pathways is crucial. A recent study sheds light on the potential consequences of R-rich DPR interaction with the nuclear import receptors (NIRs), proteins responsible for transporting molecules inside the cell nucleus through the nuclear pore complex^53^. The study revealed that NIR recruitment into DPR aggregates impairs their ability to maintain the solubility of other RNA-binding proteins (RBPs), such as FUS. Even though it is outside the scope of this study to analyze the behavior of the different RBPs, in the case of TDP-43, we did not observe TDP-43 aggregates other than the ones formed by co-aggregation with GR and Kapβ2.

The pathogenesis of C9orf72-associated ALS/FTD is likely multifactorial, involving multiple mechanisms that interact with each other. A Kapβ2-based therapeutic strategy might not bring enough benefits as a stand-alone treatment for ALS but might work in conjunction with drugs targeting different pathways. Kapβ2 levels could be raised in neurons using a gene therapy approach such as mRNA-nanoparticle technology^54^. Future studies would also be directed to screening for compounds that can induce the expression of Kapβ2 in neurons. Another intriguing therapeutic possibility is *via* promoting arginine methylation. For instance, the Kapβ2-FUS interaction is modulated by the FUS methylation status^55^. Arginine methylation is also a GR post-translational modification that sometimes dictates disease severity^56^. The most abundant GR methylation status in post-mortem tissues is the asymmetric di-methylated form, but GR can be present with symmetrical di-methylation or mono-methylation. It would be essential to expand on this concept and study how the different methylation status/conditions of GR influence Kapβ2 binding and, thus, GR toxicity. These studies could entail evaluating the ideal dosage and delivery modes of Kapβ2 and any possible adverse reactions and enduring impacts on the brain.

Some intrinsic limitations in our study should be taken into consideration when interpreting our results. We recognize that Pearson’s coefficient colocalization scores do not entirely demonstrate physical interaction between proteins, which is why we sought to prove the GR-Kapβ2 interaction with additional techniques, such as co-IP studies. We recognize that the presence of the GFP reporter moiety fused to the GR poly-dipeptide could interfere with some of the experiments conducted; for example, in co-IP experiments we reported binding of Kapβ2 to the GFP reporter moiety that we expressed in neurons as control. Therefore, we sought to further prove the GR-Kapβ2 interaction by rigorously quantifying the co-IP results and running and quantifying reverse co-IP using Kapβ2 antibodies. Overall, we believe that using established, orthogonal biochemical and imaging techniques is highly effective in demonstrating the interaction between GR and Kapβ2 in primary neurons and CNS tissue.

## Methods

### Human tissues

Human post-mortem CNS tissue from healthy controls and patients carrying the *C9ORF72* intronic nucleotide repeat expansion was obtained from the Target ALS and the Weinberg ALS center biobank. Information and demographics of patients and controls are presented in Supplementary Table 4. Human post-mortem tissues (fresh frozen) were stored at −80 ºC.

### Cell cultures

HEK293 cells were cultured in DMEM medium (Cytiva Cat.# SH30243.LS) supplemented with 10% FBS (Cytiva Cat.# SH30071.01HI), penicillin, and streptomycin (Thermo Fisher Scientific Cat.# SV30010). Cells were passaged every 3–4 days using 0.05% trypsin (Corning Cat.# 25-051-C).

### Primary cortical neurons

After meninges removal, brains were dissected from embryonic day 16 (E16) rat embryos. Cortices and midbrain regions were cut into small pieces and incubated on a shaker at 80 rcf for 45 min at 37 °C in 0.2% trypsin in HBSS without Ca^2+^ and Mg^2+^ (Cytiva Cat.# SH30588.01). FBS (Cytiva Cat.# SH30071.01HI) was added, and the cell suspension was centrifuged at 800 rcf for 10 min. at 4 °C. Cells were washed with Ca^2+^ and Mg^2+^ free HBSS and centrifuged at 800 rcf for 10 min. at 4 °C. The cell suspension was then passed through a 70 μm strainer (Foxx Life Sciences Cat.# 410-0002-OEM) to remove undigested connective tissue and large cell clumps. Dispersed cells were then counted and plated in poly-D lysine-coated plates. The following plating densities were used: 150,000 cells/well in 24 well plates, 300,000 cells/well in 12 well plates, and 5,000,000 cells/dish in 10 cm dishes. Arsenite 0.5 mM was applied for 30 min. immediately before processing the neurons for analysis. DSP (dithiobis(succinimidyl propionate)) was added to the neurons before harvesting at a concentration of 0.1 mM to the neurons in culture before processing them for western blot analysis.

### Animals

B6.C-Tg(CMV-cre)1Cgn/J::B6.C-GR50 (GR_50_) or B6.C-Tg(CMV-cre)1Cgn/J::B6.C-GFP (GFP) mice were generated as described in ref. ^6^. At 12 months, male mice were sacrificed, and cortices and spinal cords were collected and frozen in liquid N_2_ or embedded in OCT. Frozen samples were stored at −80 °C till the time of processing. All animal procedures were compliant with the ARRIVE guidelines and approved by IACUC. We have complied with all relevant ethical regulations for animal use.

### Plasmids and siRNA

The following plasmids were used: pCDNA3_hSyn_Td_Tomato; pCDNA3_hU6_Flag_mCherry; pCDNA3_hU6_Flag_GR_50__mCherry; pCDNA3_hU6_Flag_GR_100__mCherry; pCDNA3_CMV_eGFP; pCDNA3_CMV_eGFP_Kapβ2.

eGFP and eGFP_Kapβ2 were co-transfected with Td_Tomato at a DNA concentration ration of 1:2 (200 ng/400 ng) or 1:4 (200 ng/800 ng). eGFP and eGFP_Kapβ2 were co-transfected with mCherry, mCherry_GR_50_ and mCherry_GR_100_ at 1:1 ratio (400 ng/400 ng).

Human TDP-43 and TDP-43-GFP were subcloned into pE-SUMO (LifeSensors, Malvern, PA). All plasmid inserts were sequenced and confirmed to be correct.

To silence Kapβ2, 50 μM of ON-TARGETplus siRNA-TNPO1-Smart Pool (Horizon Discovery, L-086861-02-0005) was used. 50 μM of AllStars Negative Control scramble siRNA (Qiagen Cat. #1027280) was used as control.

### Transfection

Lipofectamine 2000 (Fisher Scientific Cat. #11-668-019) was used to transfect HEK cells and cortical neurons (1 μL Lipo2000/500 ng total D.N.A.). HiPerfect Transfection Reagent (Qiagen Cat. #301705) was used to transfect siRNA (8μL HiPerfect/50 nM siRNA). siRNA was transfected 24 h before plasmids transfection. For cortical neurons, Lipo/DNA or RNA mix was incubated with the neurons for 1 h, and then cell media were changed with fresh ones. For HEK293 cells, the mixture was incubated with the cells overnight.

### Lentivirus constructs production

HEK293 cells were transfected with the following plasmids: pLenti_hSyn_Flag_GR_50__eGFP, psPAX2 (Addgene Plasmid #12260), pMD2.G (Addgene Plasmid #12259). Plasmids were diluted in DMEM, and PEI MAX was added as a transfection reagent. The mix was incubated with HEK293 cells confluent at 70% for 5 h; the media was changed with a fresh one. Cells supernatant was collected 48 h after transfection and centrifuged for 10 min at 2000 rcf at 4 °C. Lentivirus Concentrator (OriGene Technologies Cat. #TR30025) was added to the supernatant and incubated overnight at 4 °C. To pellet the virus, the media was centrifuged at 500 rcf for 45 min at 4 °C. Viral particles were then resuspended in Ca^2+^ and Mg^2+^ free PBS (Cytiva Cat. #SH30028. L.S.), aliquoted and immediately frozen in liquid N_2_.

### Purification of recombinant proteins

His_6_-SUMO1 N-terminally tagged TDP-43-WT and TDP-43-GFP were purified as described (McGurk et al., 2018) and overexpressed in BL21(DE3) RIL *E.coli* cells. These cells were then sonicated on ice in 50 mM HEPES (pH 7.5), 2% TritonX-100, 300 mM NaCl, 30 mM imidazole, 5% glycerol, 2 mM β-mercaptoethanol, and protease inhibitors (cOmplete, EDTA-free, Roche). The recombinant TDP-43 proteins were purified over Ni-NTA agarose beads (Qiagen) and eluted using 50 mM HEPES (pH 7.5), 150 mM NaCl, 300 mM imidazole, 5% glycerol, and 5 mM DTT. The buffer was exchanged with the same buffer without imidazole. The eluate containing the recombinant TDP-43 proteins was then frozen in liquid N_2_ and stored as aliquots at −80 °C until use.

Kapβ2 was purified as described (ref. ^34^). In brief, *E. coli* BL21-CodonPlus(DE3)-RIL cells (Agilent) were transformed with GST-Tev-Kapβ2 plasmid, and expression was induced overnight at 25 °C with 1 mM IPTG. Cells were pelleted and resuspended in Tris buffer (50 mM Tris pH 7.5, 100 mM NaCl, 1 mM EDTA, 20% (v/v) glycerol, 2 mM DTT, supplemented with protease inhibitors), then lysed by sonication. Cell lysate was then loaded onto glutathione Sepharose^TM^ 4 Fast Flow resin (GE Healthcare) and washed with Tris buffer, followed by ATP buffer (50 mM Tris pH 7.5, 100 mM NaCl, 1 mM EGTA, 0.5 mM MgCl_2_, 5 mM ATP, 20% glycerol, 2 mM DTT, supplemented with protease inhibitors), then washed and eluted with Buffer A (20 mM imidazole, 75 mM NaCl, 1 mM EDTA, 20% (v/v) glycerol, 2 mM DTT). Finally, the protein was cleaved with Tev protease and purified on a HiTrap Q HP column (GE Healthcare) using a salt gradient. Purified protein was concentrated, flash frozen, and stored at −80 °C. Before the aggregation assay, the protein was thawed and centrifuged at 16,100 × *g* for 10 min to remove any preformed aggregates. Protein concentration was determined by Bradford assay (Bio-Rad, Hercules, CA).

Equimolar concentrations of Kapβ2, TDP-43, and GR_20_ (5μM) were used. 200 nM HIS_6_-SUMO-TDP-43-GFP and 100 nM TAMRA-GR_20_ were added to visualize TDP-43 and GR_20_.

### Peptides

Chemically synthesized GR_20_ and Tetramethylrhodamine (TAMRA)-tagged GR_20_ were obtained as a lyophilized powder from Peptide2.0 and dissolved in PBS.

### Sample preparation

100 mg of fresh frozen lumbar spinal cord samples were homogenized in 1% SDS using a 15 ml Dounce Homogenizer. The homogenate was centrifuged at 3000 rcf for 20 min at 4 °C to remove tissue debris. Protein estimation was performed on the clear homogenate using a BCA assay (Pierce BCA kit #23225). 30 μg of protein samples are loaded for Western blot analysis.

For mouse tissues: 50 mg of CNS tissues were chopped into small pieces, incubated in RIPA for 2 h, and homogenized by pipetting every 20/25 min. Samples were then sonicated using Bioruptor Pico-Diagenode.

For cell cultures: Cells were harvested in PBS and centrifuged at 500 rcf for 3 min. PBS was removed by suction, and samples were resuspended in 80 μL of RIPA with protease inhibitor (Sigma-Aldrich Cat# P2714-1BTL). Cells were incubated for 15 min at 4 °C on an orbital shaker and then sonicated. After sonication, samples were centrifuged at 16,000 rcf for 5 min at 4 °C. Supernatant was kept, and BCA was used to measure the protein content of each sample.

### Western blot

Cell cultures and animal tissues: 15 μg of protein extracts were loaded on pre-cast Stain Free gels (Bio-Rad Cat.#4568034) and run for 1 h at 100 V. Gels were then transferred on PVDF membrane (Millipore Cat.#IPVH00010) overnight at 4 °C at 30 V and developed with primary and secondary antibodies at the indicated concentration. Before transfer, gels were cross-linked by UV light exposure for 5 min, and total protein content was then revealed on the membrane by UV light exposure. Membranes were developed with SuperSignal™ West Femto Maximum Sensitivity Substrate (Thermo Fisher Scientific Cat#34094) and imaged using ChemiDOC XRS+ System (Bio-Rad).

Human samples: The gels were U.V. activated for 5 min before transferring onto the 0.22 μm nitrocellulose membrane. The semi-dry transfer was done using a Trans-Blot Turbo Transfer System (Bio-Rad) at 25 mV for 10 min. After the transfer, a stain-free blot image was captured and used as total protein content. The blot was processed as previously described. The quantification was done using Image Lab Software (Bio-Rad); the intensity of the Kapβ2-positive band is normalized to its total proteins. Quantification was performed using Image Lab software. Adjusted volume was measured for each band and divided by the adjusted volume calculated, considering the entire lane of total proteins. The abundance of the protein of interest was normalized to the total amount of proteins in each lane, therefore removing variations associated with comparing abundance to a single protein arbitrarily chosen as housekeeping protein (e.g., GAPDH). Total protein normalization is more compatible with detecting and quantifying proteins of lower abundance.

### Immunoprecipitation

Flag: Immunoprecipitation was carried out using anti-Flag agarose beads (mouse IgG agarose beads were used as control). Rat primary cortical neurons and tissues were extracted in IP buffer containing 50 mM Tris-HCl (pH 7.5), 150 mM NaCl, 1 mM EDTA, 0.5% NP-40, 100 mM EDTA (pH 8.0) supplemented with protease inhibitor (Sigma-Aldrich Cat.# P2714-1BTL). After incubating at 4°C for 30 min., the samples were sonicated and then centrifuged for 10 min. at 10,000 rcf. Bradford assay was used to determine protein concentration. The agarose beads were added to 600 μg of cell lysates and incubated at 4 °C for 24 h under constant shaking. Cell lysates were centrifuged for 1 min. at 1000 rcf to collect the supernatant. The pellets were washed five times, but only the first wash was collected. After the last centrifugation, the samples were eluted with SDS-PAGE loading dye at 95 °C for 5 min. The entire eluate was run for Western blot. 2% of total lysate was used as input.

GFP and Kapβ2: Following manufacturer instructions, immunoprecipitation was carried out using magnetic beads (Thermo Fisher Scientific Cat.# 88804). Briefly, 25 μL of magnetic beads were conjugated with 10 μg of the desired antibody overnight and then crosslinked for 30 min using DSS (disuccinimidyl suberate). The obtained beads-antibody complex was incubated with cell or tissue lysates overnight. The next day, beads were washed, and the bound protein was eluted. The entire eluate was run for Western blot. 2% of total lysate was used as input. For Kapβ2 IP, a double volume of beads was used.

Quantification: signal specifically corresponding to Kapβ2 and GFP bands was measured with ImageLab and the ratio was calculated between the respective adjusted volumes.

### Immunofluorescence

Primary cortical neurons were fixed for 20 min at 37 °C with 4% PFA and incubated in PermBlock solution (2% BSA, 0.3% Triton-x, 5% donkey serum in PBS). Primary antibodies were diluted in 0.1% BSA cells and were incubated overnight at 4 °C on an orbital shaker. The following day, cells were washed two times with PBS at room temperature for 10 min and incubated with the appropriate secondary antibody for 1 h at room temperature. Cells were washed twice in PBS and incubated with Hoechst solution (Thermo Fisher Scientific Cat.# 62249) for 10 min at room temperature to stain the nuclei. After a final rinse in PBS, coverslips were mounted on glass slides using AquaMount (Lerner Laboratories Cat.# 13800).

Tissues were cryo-sectioned using Cryostar NX50. 20 μm thick sections were placed on glass slides and incubated for 10 min in 4% PFA. Slides were then washed in TBS-T for 10 min and incubated for 1 h at room temperature in PermBlock solution (2% BSA, 0.3% Triton-x, 5% donkey serum in PBS) and then incubated with primary antibody diluted in 1% BSA, 0.3% Triton-x and 1% donkey serum overnight at 4 °C in a humidified chamber. The following day, slides were washed with TBS-T, incubated with the appropriate fluorescent secondary antibody for 1 h at room temperature, rinsed twice in TBS-T, incubated for 10 min with Hoechst (Thermo Fisher Scientific Cat.# 62249), and mounted on coverslips using AquaMount (Lerner Laboratories Cat.# 13800) for imaging analysis.

### Antibodies and dilutions

Primary antibodies: anti-Kapβ2 (RRID:AB_2206884, Santa Cruz Biotechnology, Inc Cat.# sc-101539; WB dilution 1:1000); anti-Kapβ2 (RRID:AB_262123, Sigma Cat.# T0825; IF dilution 1:500); anti-Map2 (RRID:AB_2138178 Novus Cat.#NB300-213; IF dilution 1:2000); anti-GFP (RRID:AB_11042881, Proteintech Cat.#50430-2-AP, WB dilution 1:1000); anti-TDP-43 (RRID:AB_615042, Proteintech Cat.#10782-2-AP; IF dilution 1:500); anti-GAPDH (RRID:AB_2107436, Proteintech Cat.#60004-1-Ig; WB dilution 1:1000); Neun (D4G40) XP(RRID:AB_2651140, Cell Signaling Technology Cat.#24307; IF dilution 1:500): FUS (RRID:AB_2247082, Proteintech Cat.#11570-1-AP; IF:1:500).

Secondary HRP-conjugated antibodies: HRP-Conjugated anti-rabbit (RRID:AB_772191, Cytiva Cat.# NA9340-1ML; WB dilution 1:10,000); HRP-Conjugated anti-mouse (RRID:AB_772193, Cytiva Cat.# NA9310-1ML; WB dilution 1:10,000); HRP-Conjugated anti-rat (RRID:AB_2936877, Sigma Cat.# AP136P; WB dilution 1:3,000);

Secondary fluorescent antibodies: AlexaFluor-546 anti-mouse (RRID:AB_2534012, Thermo Fisher Scientific Cat.# A10036; IF dilution 1:1,000); AlexaFluor-647 anti-rabbit (RRID:AB_2536183, Thermo Fisher Scientific Cat.# A31573; IF dilution 1:1,000); AlexaFluor-647 anti-chicken (RRID:AB_2762845, Thermo Fisher Scientific Cat.# A32933; IF dilution 1:1,000);

### Extraction of total RNA and quantitative Real-Time PCR

Total RNA was extracted using Trizol Reagent (Ambion Cat.# 15596026) following the manufacturer’s instructions. Briefly, cells were lysed in Trizol Reagent and homogenized using a syringe for insulin. RNA was then extracted in isopropanol. Samples were centrifuged for 15 min. at 12,000 rcf. The RNA-containing pellet was washed with 75% ethanol and centrifuged for 5 min. at 7500 rcf. Dried pellets were resuspended in DEPC water, and concentration was assessed with Nanodrop. 500 ng of total RNA was first treated with DNAse I (Thermo Fisher Scientific Cat.# EN0521) and retrotranscribed using SuperScript™ IV Reverse Transcriptase (Thermo Fisher Scientific Cat.# 18090010). The cDNA was diluted 1:2 and used for qRT-PCR, which was performed using the following TaqMan assay: GAPDH-VIC (Thermo Fisher Scientific Cat# 4352338E) and Kapβ2-FAM (Thermo Fisher Scientific Cat.# 4331182 Assay# Rn01489969_m1). 50 ng of cDNA was used per reaction. Samples were analyzed using the Design and Analysis tool by Thermo Fisher Scientific. Results are shown as fold change using the 2^ΔΔCt^ method.

### Confocal imaging analysis

Confocal images were taken on a Nikon A1R confocal microscope with a 60X objective. Z-stacks were taken for each image. 0.2 μm Z-stacks were taken to perform colocalization studies and puncta counting using a built-in function in Imaris. 1 μm stacks were taken for TDP-43 nuclear intensity studies. Maximum intensity projection was then generated through a built-in function of the A1R analysis software. The nuclei of each co-transfected cell were selected as the region of interest, and TDP-43 intensity was assessed. The mask was made on the mCherry channel to analyze aggregate content, and the TDP-43 fluorescence was subsequently evaluated. For Kapβ2 nuclear and cytoplasmic quantification maximum intensity projections were generated, and ROI were drawn around nucleus and cytoplasm and intensity referred to the Kapβ2 chnnel was measured. The ratio between nuclear and cytoplasmic intensities in the same cell was then calculated.

FRAP experiment was performed in green (GFP or GFP-Kap2) and red (mCherry, GR50-mCherry, GR100-mCherry) fluorescent cells. Aggregates were selected as regions of interest and photobleached for 2 s. with blue light with laser power at 25% power. Following photobleaching, images were taken every 2 s for 3 min and analyzed with Nikon A1R software. Normalization and curve fitting were performed using the online tool EasyFRAP (normalization was performed using double normalization, and curve fitting was performed using double equation). The fluorescence recovery intensity curves were represented over time using Prism GraphPad 9.0. The mobile fraction in FRAP experiments represents the proportion of molecules or particles that can freely move within the sample after photobleaching. The mobile fraction was calculated using the following formula: Mobile Fraction = Mobile Fluorescence Recovery\Initial Fluorescence Loss where the mobile fluorescence recovery is the increase in fluorescence signal after photobleaching, indicating the recovery of mobile molecules, and the initial fluorescence Loss is the initial decrease in fluorescence signal due to photobleaching.

### Time-lapse microscopy

Cells were imaged every 2 days after transfection. 20× objective was used, and 25 fields/wells were acquired. Only cells positives for both Kapβ2 and mCherry were counted. Cells were scored dead when they disappeared from the field of view or clear signs of neurite fragmentation appeared. Kaplan Meier curves were then constructed in Prism GraphPad 9.0.

### Visualization of TDP-43 Condensates

His_6_-SUMO1-TDP-43 (5 μM) was incubated with an equimolar concentration of (GR)_20_ for 1 h at room temperature in PBS. 200 nM His_6_-SUMO1-TDP-43-GFP and 100 nM TAMRA-(GR)_20_ were added to visualize TDP-43 and (GR)_20_. The reaction mixture was incubated at room temperature for 1 h, and samples were spotted onto a coverslip and imaged by DIC and fluorescent microscopy using a Leica DMi8 Inverted microscope. To inhibit condensation, 5 μM Kapβ2 was added at the beginning of the assay.

### Proteomics analysis

Publicly available data sets from ref. ^44^. were downloaded from massive.ucsd.edu (MassIVE MSV000088581). Analysis and statistical comparison of protein abundance were performed using basic packages (ggplot, ggplot2) of R (version 4.0.5) and Rstudio (version 2022.07.2).

### Transcriptomics analysis

In this study, we performed a transcriptomic analysis using publicly available datasets to investigate Kapβ2 expression patterns in iPS-derived cortical neurons and ALS patients’ cerebral tissue.

For the Answer ALS data: The raw sequencing data were processed using quality control metrics and aligned to a reference genome using Kallisto. Differential gene expression analysis was performed using DESeq2. Kapβ2 expression levels across samples were analyzed with ggplo2, and statistical analysis was carried out using GGPUBR.

For the human tissue data, raw counts were downloaded from https://github.com/NathanSkene/ALS_Human_EWCE. Kapβ2 expression levels across samples were analyzed with ggplo2; statistical analysis was done using GGPUBR.

### Statistics and reproducibility

All experiments were performed at least 3 times (n ≥ 3). At least three mice/group were used. For live-cell imaging, >150 neurons/condition were counted for each n. For colocalization, puncta counting, and TDP-43 intensity, ≥10 neurons/condition were counted for each n. For the FRAP experiment, ≥5 cells/conditions were analyzed for each n. When two groups were compared, the Kolmogorov-Smirnov test assessed the normality of each population. If the two populations’ results were normally distributed, then the unpaired t-student test was performed using the built-in function in Prism GraphPad 9.0. If one or both populations were not normally distributed, then the Mann-Whitney test was used to compare ranks or the Kolmogorov-Smirnov test was used using Prism GraphPad 9.0. The log-rank and Cox proportional hazards tests compared the Kaplan Meier curves and the statistical significance between curves was assessed with Cox (GraphPad software). For the FRAP experiment, statistical differences between the curves were evaluated by curve analysis (one-way ANOVA test) in Prism GraphPad 9.0.

### Reporting summary

Further information on research design is available in the Nature Portfolio Reporting Summary linked to this article.

### Supplementary information

Supplementary Information

Description of Supplementary Materials

Supplementary Movie 1

Supplementary Movie 2

Reporting Summary

## Data Availability

The data that support the findings are available at the following 10.5061/dryad.v41ns1s4f All uncropped blots are presented in Supplementary Fig. 8. Deposited Data from Liu et al., MSV000088581. Deposited Data from Manberg et al., https://raw.githubusercontent.com/NathanSkene/ALS_Human_EWCE/master/Data/Raw/GSE67196_Petrucelli2015_ALS_genes.rawcount_EXP.csv. Deposited Data for analysis of iPS derived cortical neurons come from the Answer ALS repository https://dataportal.answerals.org/home. All the data are available from the corresponding author upon request.

## Code availability

Custom codes were used to analyze transcriptomics data from public repositories using R studio. These codes are available at the following 10.5061/dryad.v41ns1s4f.
