# Supplementary material for: The nuclear import receptor Kapβ2 modifies neurotoxicity mediated by poly(GR) in C9orf72-linked ALS/FTD
Source: Commun Biol. 2024 Mar 28;7:376. doi: 10.1038/s42003-024-06071-2 (PMC10978903; doi:10.1038/s42003-024-06071-2)
Supplement: Supplementary file 1 — Supplementary Information [file 42003_2024_6071_MOESM1_ESM.pdf]

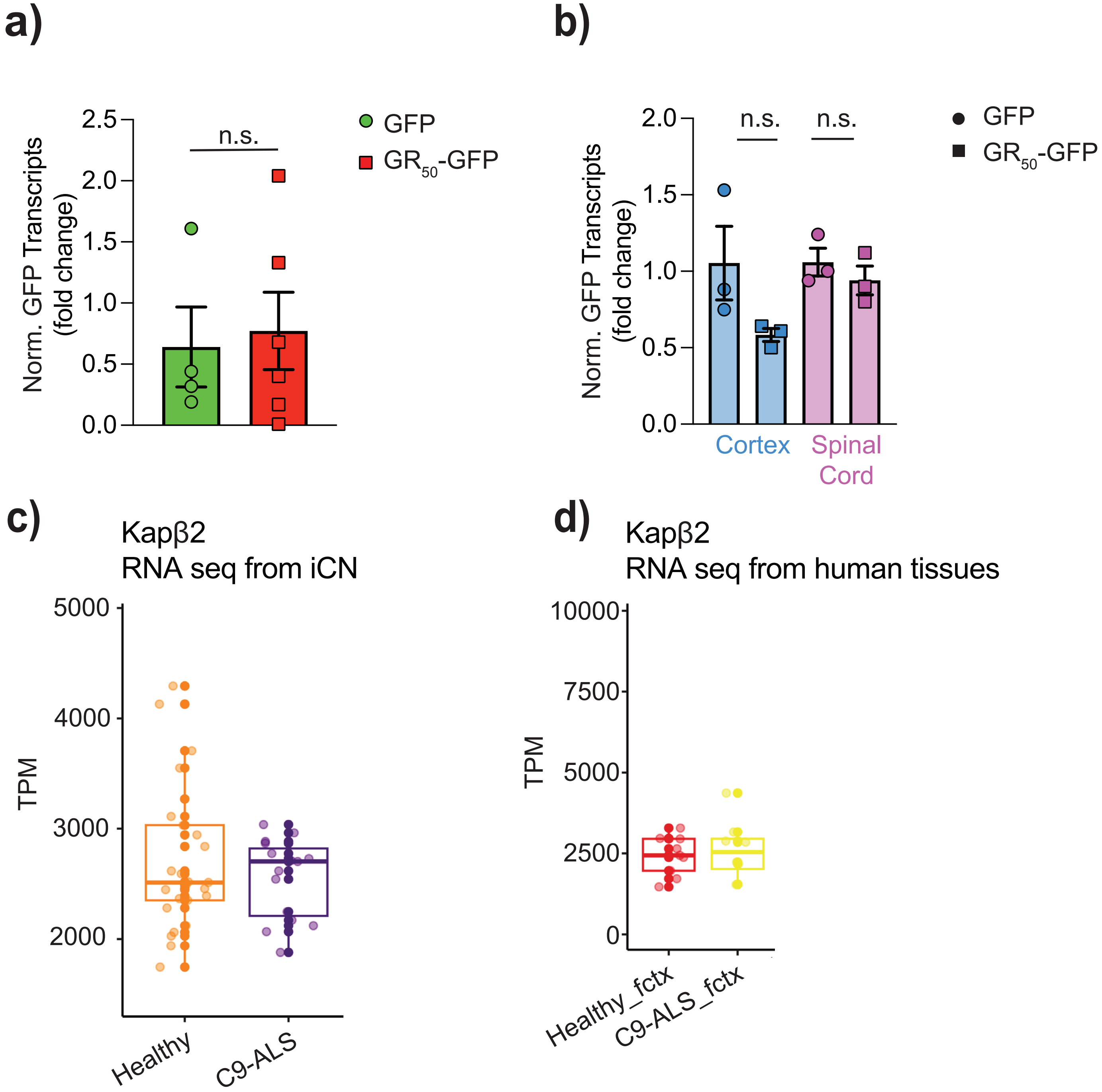

Data Source: AnswerALS

Data Source: Manberg et al., PMCID: PMC7613336

Supplementary Figure 1

a) qPCR analysis of GFP transcript levels in cortical neurons transduced with GFP or GR_{50}. Data are represented as mean ± S.E.M. (n=6 biological replicates, Student t-test, n.s.=not significant)

b) qPCR analysis of GFP transcript levels in the cortex and spinal cord of GFP and GR_{50} mice. Data are represented as mean ± S.E.M. (n=3 biological replicates, One Way-ANOVA, n.s.=not significant)

c) Box plot showing Kapβ2 transcript levels in iPS-derived cortical neurons (iCN) from control (healthy) and C9orf72 (C9-ALS) patients. (n>15 biological replicates, Student t-test, n.s.=not significant)

d) Box plot showing Kapβ2 transcript levels in the frontal cortex (fctx) of control (healthy) and C9orf72 (C9-ALS)patients (n>10 biological replicates, Student t-test, n.s.=not significant).

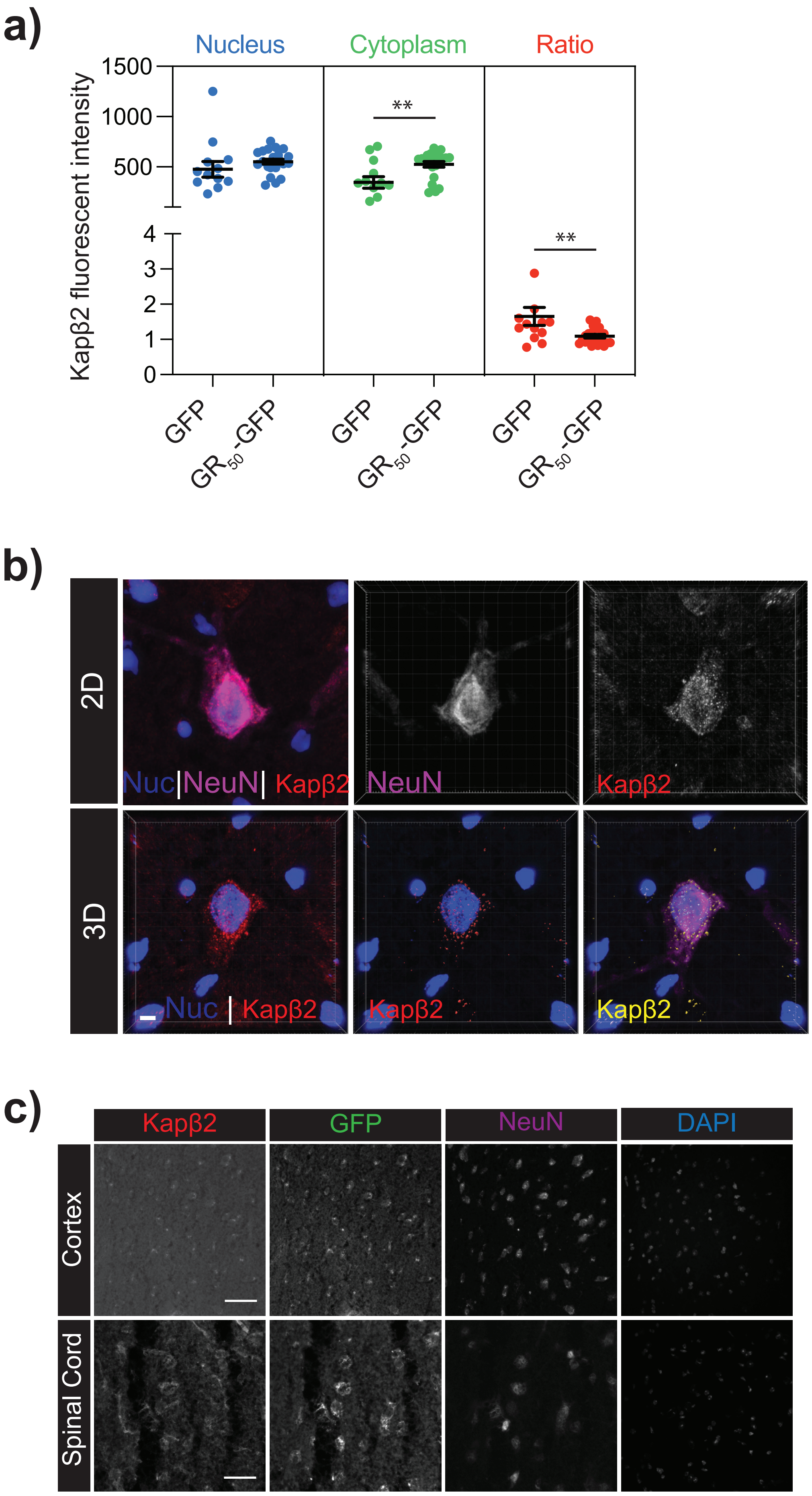
a)
Nucleus
Cytoplasm
Ratio
Kapβ2 fluorescent intensity
1500
1000
500
4
3
2
1
0
**
**
GFP
GR50-GFP
GFP
GR50-GFP
GFP
GR50-GFP
b)
2D
3D
Nuc|NeuN| Kapβ2
NeuN
Kapβ2
Nuc | Kapβ2
Kapβ2
Kapβ2
c)
Kapβ2
GFP
NeuN
DAPI
Cortex
Spinal Cord

Supplementary Figure 2

a) Quantification of Kapβ2 nuclear and cytoplasmic fluorescence intensity in cortical neurons transduced with GFP or GR_{50}-GFP and their ratio. Data are represented as mean ± S.E.M. (n=3 biological replicates, m>3 neurons, Student t-test, n.s.=not significant)

b) Upper inset: confocal imaging of WT mouse spinal cord. Red is Kapβ2; far red is NeuN. Lower inset Imaris rendering of Kapβ2 staining in WT mouse spinal cord.

c) Confocal images of Figure 2c presented as individual fluorescence channels not in pseudo-color. Scale bar = 30μm

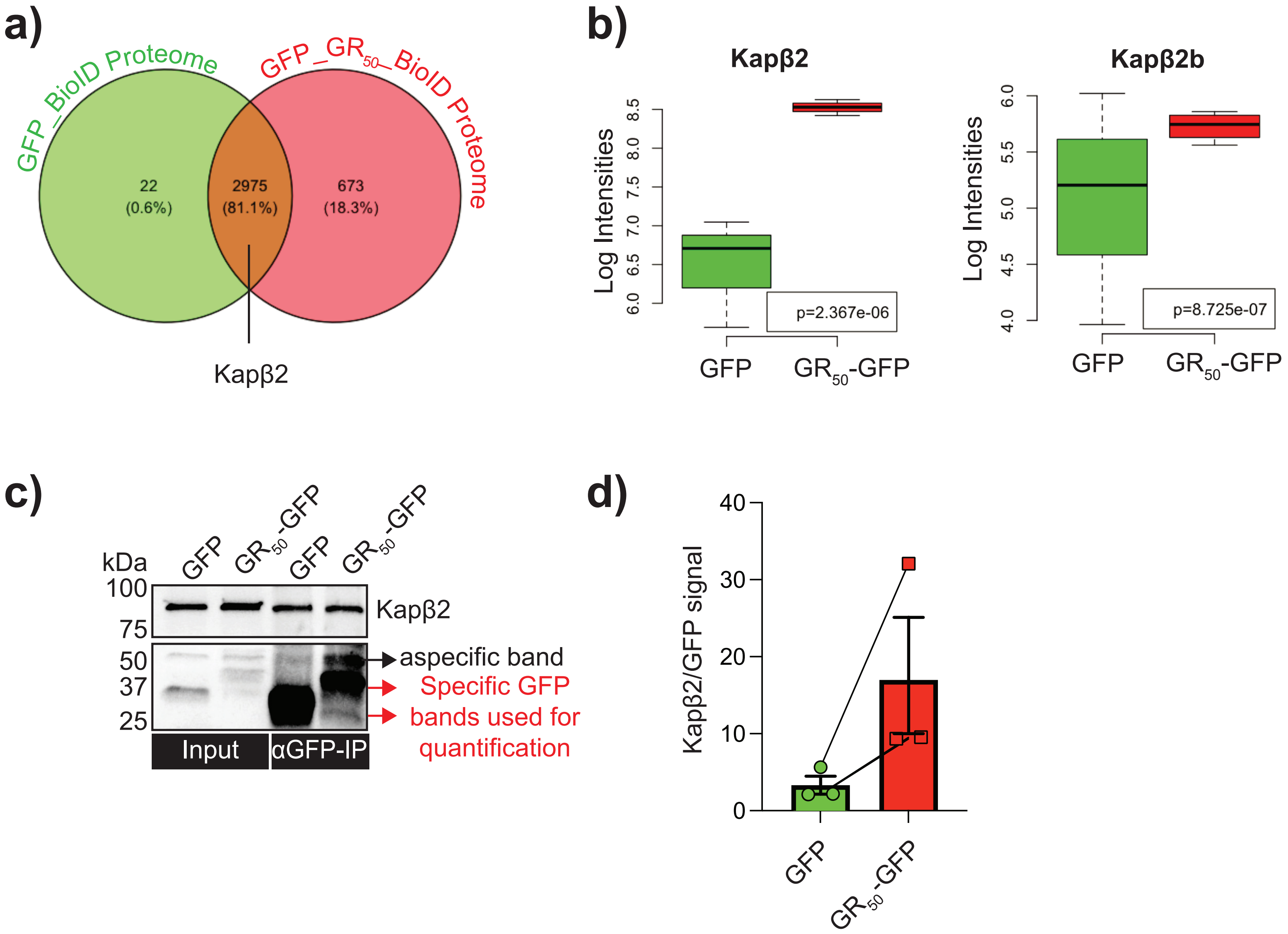

a)
GFP_BioID Proteome
GFP_GR50_BioID Proteome
22 (0.6%)
2975 (81.1%)
673 (18.3%)
Kapβ2
b)
Kapβ2
Kapβ2b
Log Intensities
p=2.367e-06
p=8.725e-07
GFP
GR50-GFP
c)
kDa
GFP
GR50-GFP
100
75
50
37
25
Kapβ2
aspecific band
Specific GFP bands used for quantification
Input
αGFP-IP
d)
Kapβ2/GFP signal
40
30
20
10
0
GFP
GR50-GFP

Supplementary Figure 3

a) Venn diagram showing the protein interacting with GFP and GR_{50}-GFP

b) The floating bar graph shows the quantification of the intensities of the peptides for Kapβ2 and Kapβ2b in GFP and GR_{50}-GFP.

c) Western blot of the immunoprecipitation assay performed on GFP and GR_{50}-GFP transduced cortical neurons. Blots were probed for GFP and Kapβ2 and total protein was used as loading control.

d) Quantification of the ratio between Kapβ2 and GFP signal from Suppl Fig 3C. Data are represented as mean $\pm$ S.E.M. (n=3 biological replicates, Student t-test, n.s.)

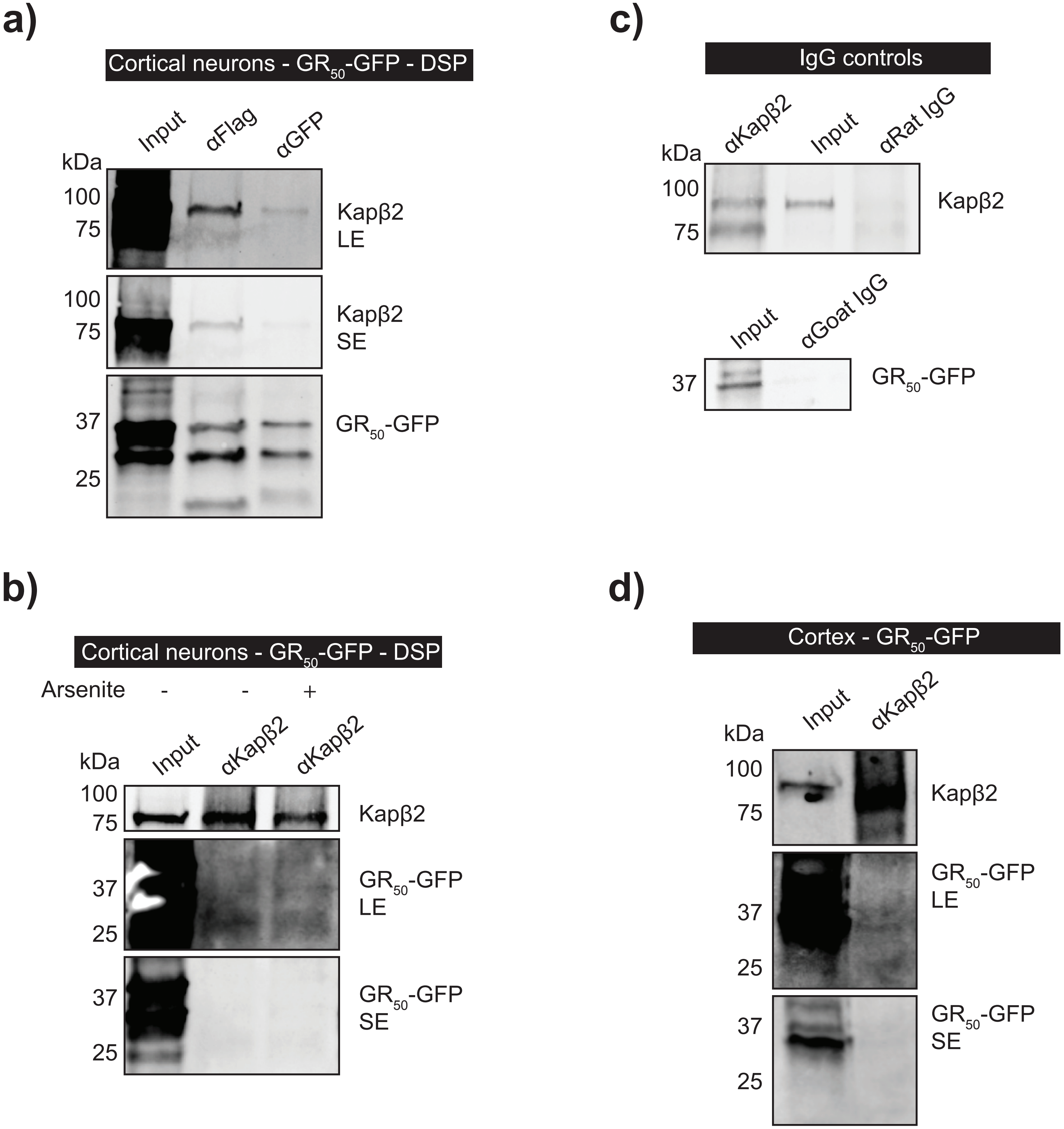
a)
Cortical neurons - GR50-GFP - DSP
Input
αFlag
αGFP
kDa
100
75
Kapβ2
LE
100
75
Kapβ2
SE
37
25
GR50-GFP
b)
Cortical neurons - GR50-GFP - DSP
Arsenite - - +
Input
αKapβ2
αKapβ2
kDa
100
75
Kapβ2
37
25
GR50-GFP
LE
37
25
GR50-GFP
SE
c)
IgG controls
αKapβ2
Input
αRat IgG
kDa
100
75
Kapβ2
Input
αGoat IgG
37
GR50-GFP
d)
Cortex - GR50-GFP
Input
αKapβ2
kDa
100
75
Kapβ2
37
25
GR50-GFP
LE
37
25
GR50-GFP
SE

Supplementary Figure 4

a) Western blot of the immunoprecipitation assay performed on GR_{50}-GFP transduced cortical neurons. The same input was processed with Flag-Beads, GFP-beads, and Kapβ2 beads. Blots were probed for GFP and Kapβ2. Total proteins were used as the loading control. SE = short exposure time, LE = long exposure time.

b) Western blot of the immunoprecipitation assay performed on cortical neurons transduced with GR_{50}-GFP virus. The input was processed with Kapβ2 beads. Blots were probed for GFP and Kapβ2. Total proteins were used as the loading control. SE = short exposure time, LE = long exposure time.

c) Western blot of the immunoprecipitation assay performed on the cortex of GR_{50}-GFP mice with anti-goat and anti-rat Igg control. Upper panel: The same input was processed with Kapβ2 beads and anti-rat Igg. Blot were probed for Kapβ2. Lower panel: The input was processed with anti-goat Igg. Blot were probed for GFP. Total proteins were used as the loading control.

d) Western blot of the immunoprecipitation assay performed on the cortex of GR_{50}-GFP mice. The same input was processed with Kapβ2 beads. Blots were probed for GFP and Kapβ2. Total proteins were used as the loading control. SE = short exposure time, LE = long exposure time.

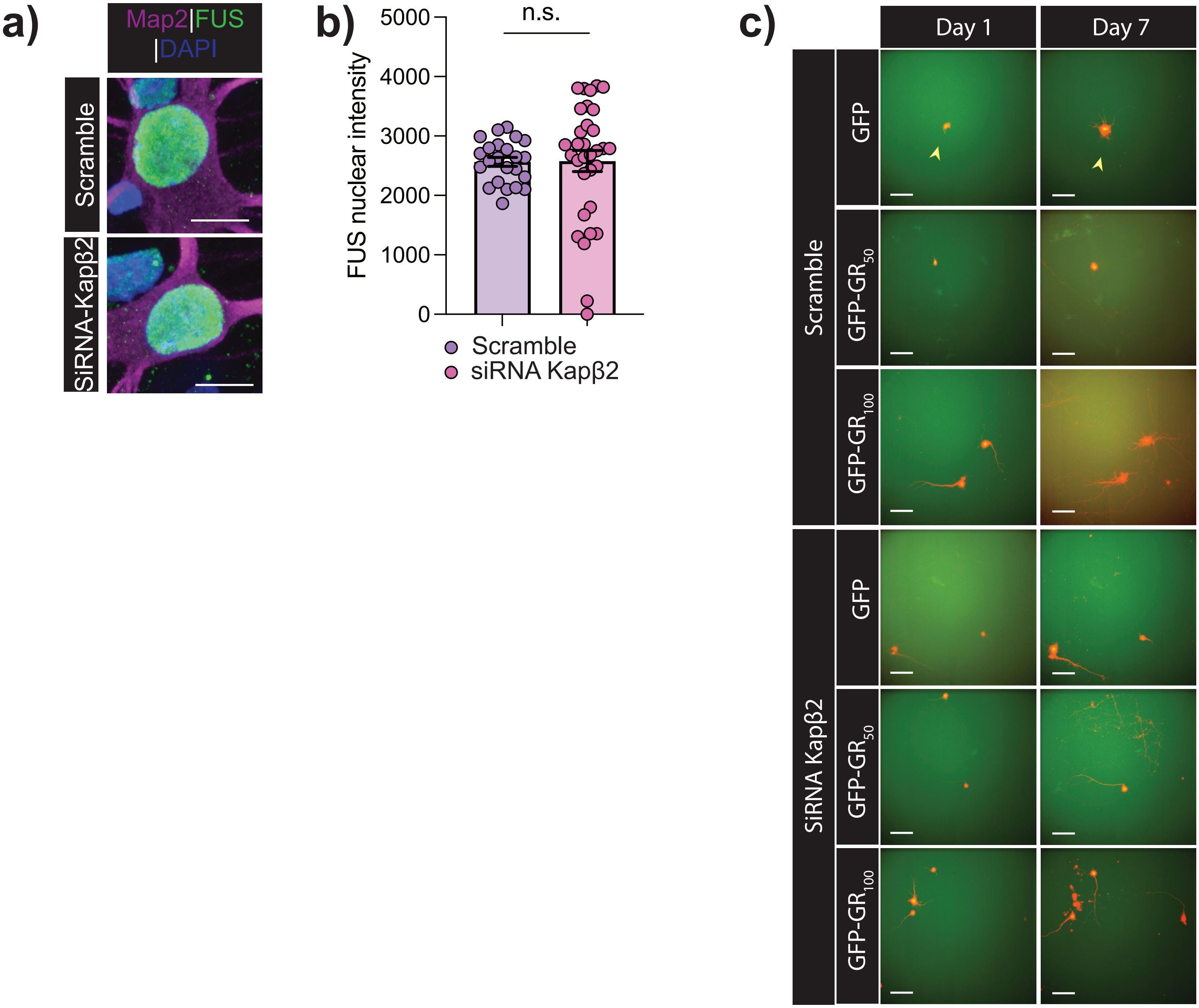
a)
Map2|FUS |DAPI
Scramble
SiRNA-Kapβ2
b)
n.s.
FUS nuclear intensity
5000
4000
3000
2000
1000
0
Scramble
siRNA Kapβ2
c)
Day 1
Day 7
Scramble
SiRNA Kapβ2
GFP
GFP-GR50
GFP-GR100

Supplementary Figure 5

a) Confocal images of primary rat cortical neurons treated with Kapβ2 or scramble siRNA (100 μM). Green is FUS, magenta is Map2, and blue is Hoechst. Scale Bar = 5μm.

b) Quantification of nuclear intensity of FUS. Data are presented as mean ± S.E.M. (n=3 biological replicates, m>20 neurons/group, Student t-test, n.s.=not significant)

c) Sample live microscopy images captured over time through epifluorescence microscopy of neurons transfected with GFP, GR_{50}-GFP, or GR_{100}-GFP and treated with Kapβ2 or scramble siRNA (100 μM)

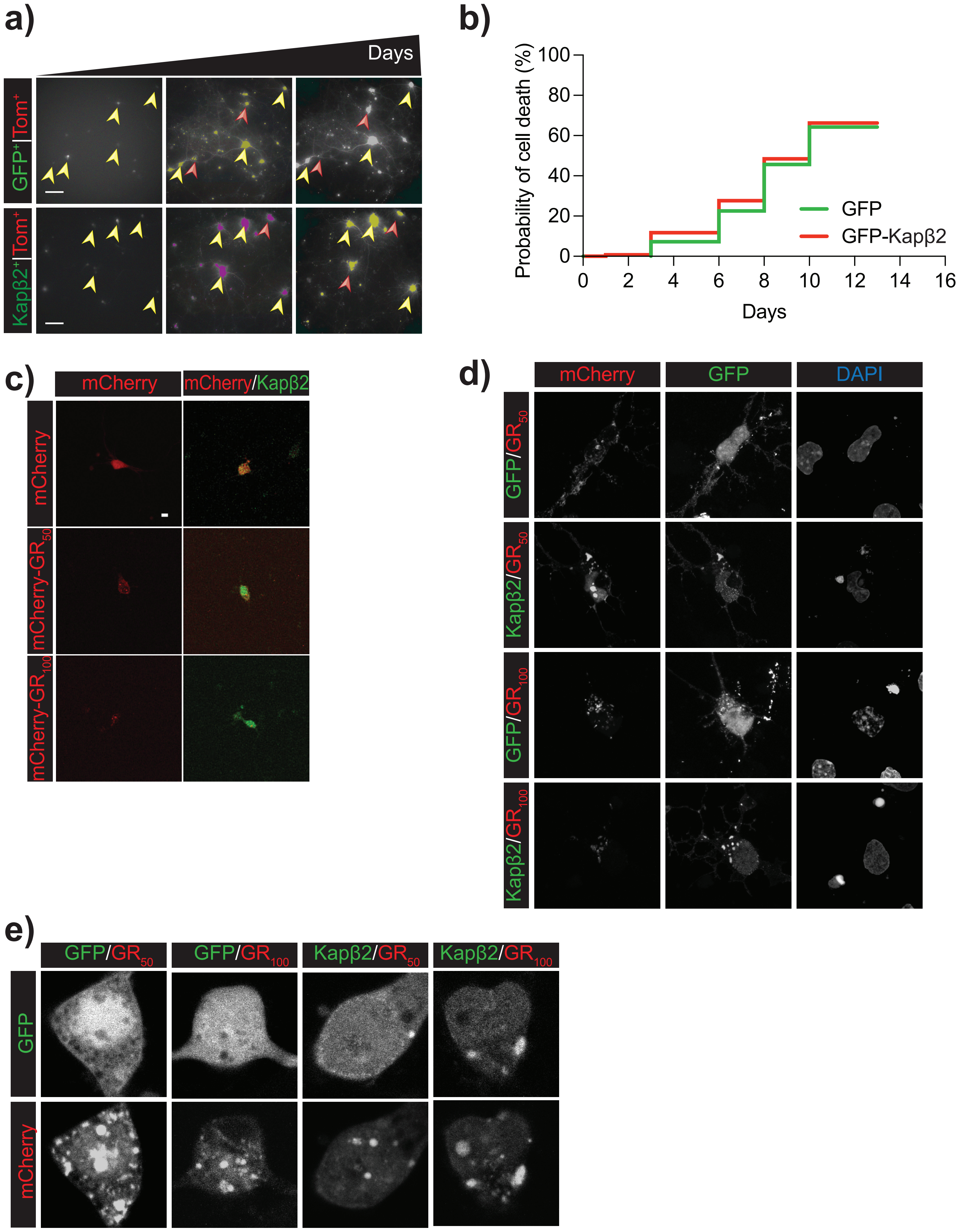
a)
Days
GFP+|Tom+
Kapβ2+|Tom+
b)
Probability of cell death (%)
100
80
60
40
20
0
0
2
4
6
8
10
12
14
16
Days
GFP
GFP-Kapβ2
c)
mCherry
mCherry/Kapβ2
mCherry
mCherry-GR50
mCherry-GR100
d)
mCherry
GFP
DAPI
GFP/GR50
Kapβ2/GR50
GFP/GR100
Kapβ2/GR100
e)
GFP/GR50
GFP/GR100
Kapβ2/GR50
Kapβ2/GR100
GFP
mCherry

Supplementary Figure 6

a) Representative images from epifluorescence live microscopy of neurons transfected with 200ng/400ng of Tm+/GFP+ and followed over time. Green is Kapβ2; red is Td-Tomato. Scale bar 20μm.

b) Probability of cell death via a cumulative risk of death plot of rat primary cortical neurons transfected with 200ng/800ng of Tm^+/GFP-Kapβ2^+ (GFP used as control) per 150,000 cells. Neurons double positive Tm^+/GFP^+ were counted (n=3 biological replicates, m>150 neurons, Log-rank Mantel-Cox test: not significant)

c) Representative images of double positive neurons transfected with 400ng/400ng of GFP-Kapβ2^+ (GFP alone used as control) and GR_{100}-mCherry GR_{50}-mCherry (mCherry alone used as control) over time. Scale bar = 10 μm.

d) Confocal images of Figure 5a presented as individual fluorescence channels not in pseudo-color. Scale bar = 10 μm.

e) Confocal images of Figure 6a, b, d, e presented as individual fluorescence channels not in pseudo-color. Scale bar = 10 μm.

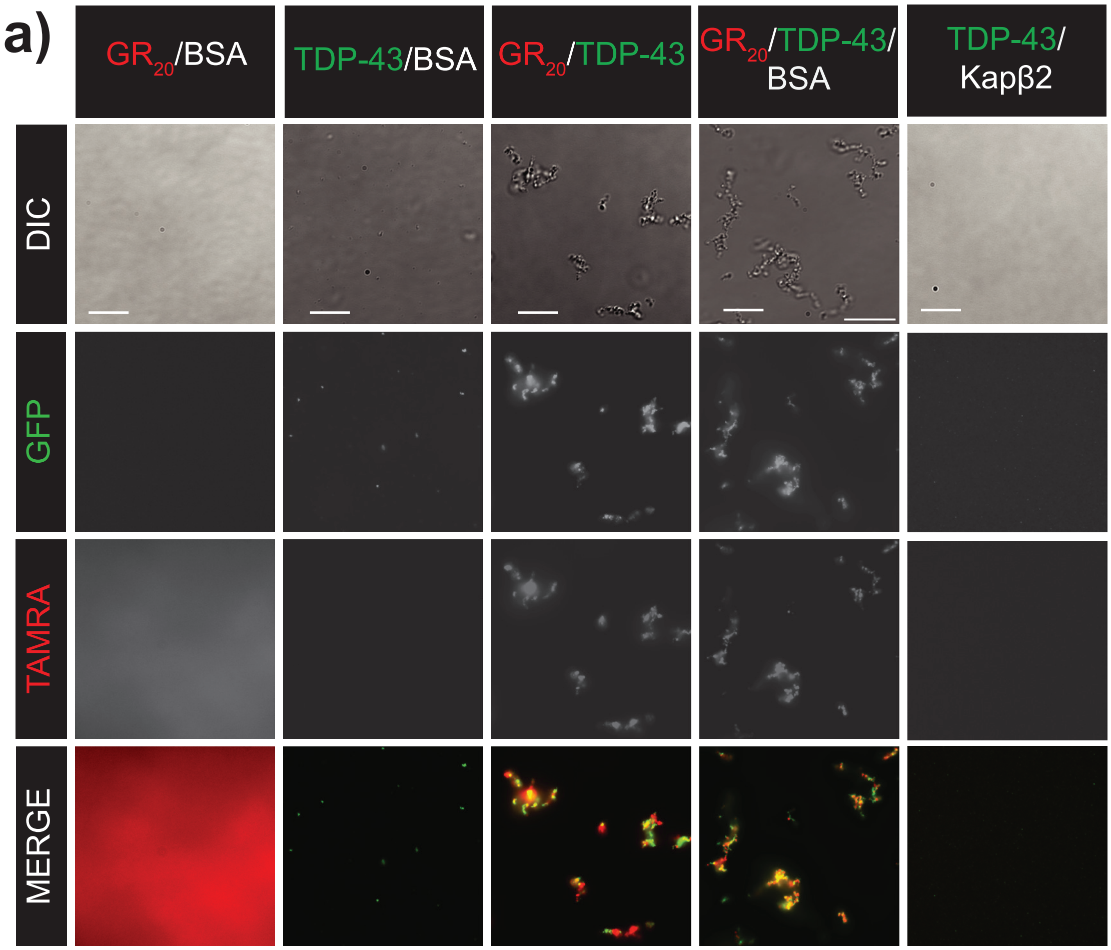
a)
GR20/BSA
TDP-43/BSA
GR20/TDP-43
GR20/TDP-43/BSA
TDP-43/Kapβ2
DIC
GFP
TAMRA
MERGE

Supplementary Figure 7

a) Equimolar concentrations (5μM) of SUMO-TDP-43, GR_{20}, TNPO1, or BSA (Thermo Scientific, Catalog 23209) were incubated in a buffer containing 1X PBS. 200nM HIS_6-SUMO-TDP-43-GFP and 100nM TAMRA-GR_{20} were added for visualization of TDP-43 and GR_{20}. The reaction mixture was incubated at room temperature for 1h. The samples were spotted onto a coverslip and imaged by Differential interference contrast (DIC) microscopy and fluorescent microscopy using a Leica DMi8 Inverted microscope. Scale bar = 15μm.

Uncropped gels - Figure 1B

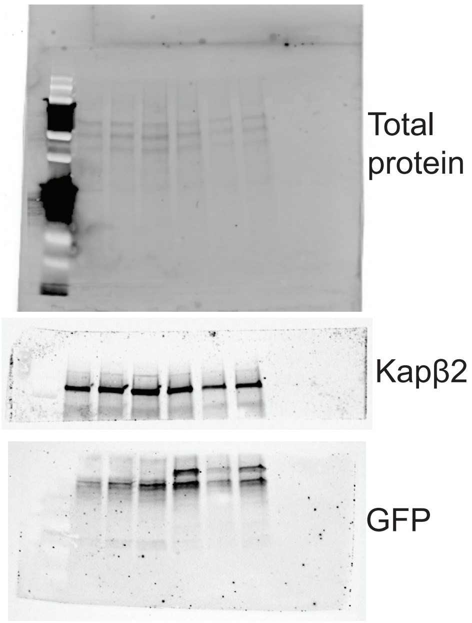

Uncropped gels - Figure 1E

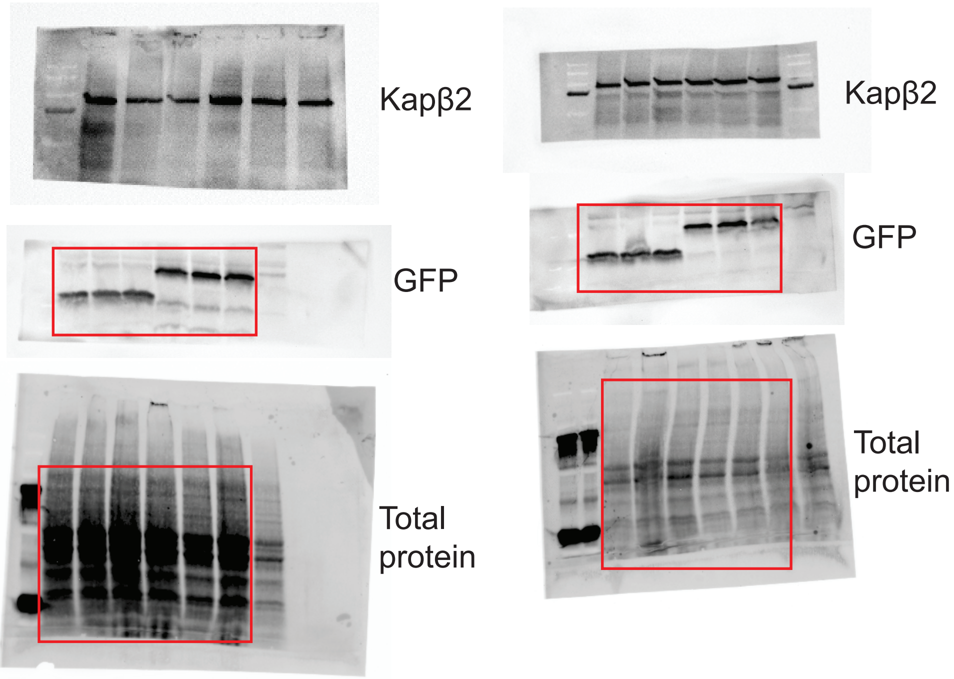

Uncropped gels - Figure 1E

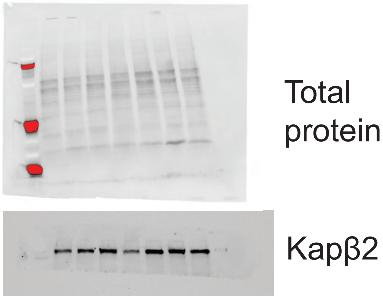

Uncropped gels - Figure 2E

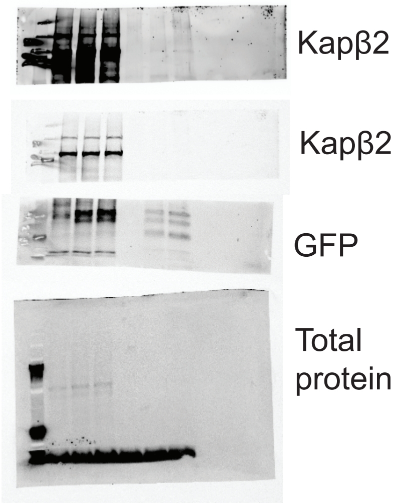

Uncropped gels - Figure 2F

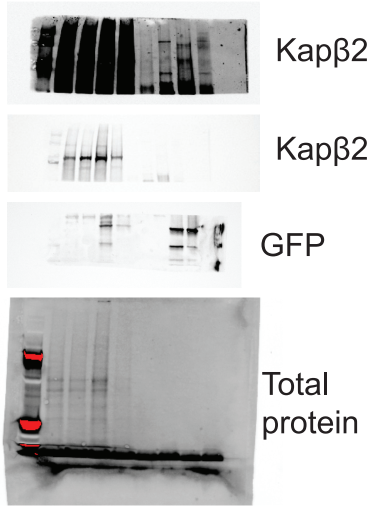

Uncropped gels - Figure 3A

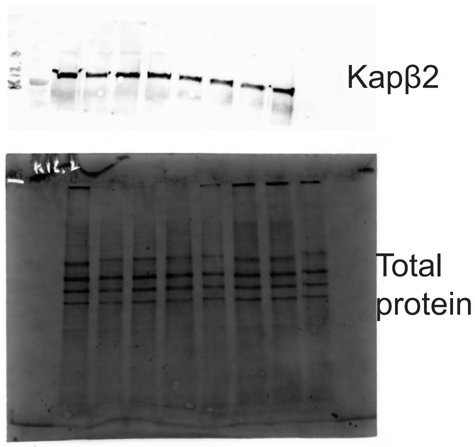

Uncropped gels - Suppl Figure 4A

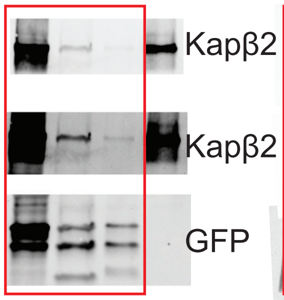

Uncropped gels - Suppl Figure 4B

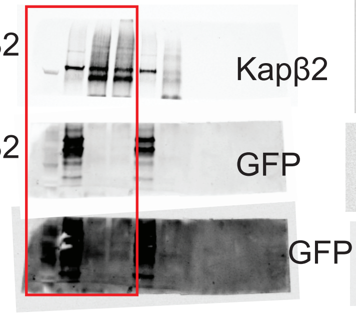

Uncropped gels - Suppl Figure 4D

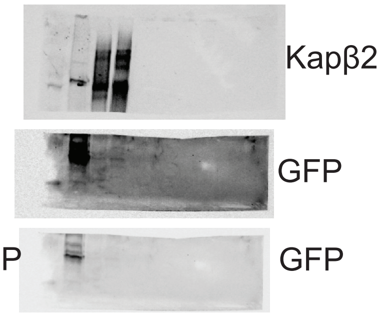

Uncropped gels - Suppl Figure 3C

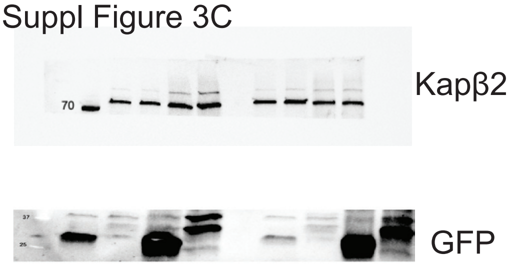

Uncropped gels - Suppl Figure 4C

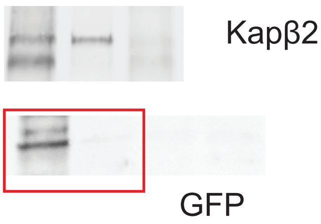

Supplementary Figure 8

Uncropped gels for western blots

**Supplementary Table 1**	# cells	p-value	Hazard Ratio	95% CI
GFP	222	0.0005	1.667	1.248 to 2.227
GR50-GFP	188	Reference Group		
GR50-GFP siRNA Kapβ2	209	0.0137	0.7068	0.5363 to 0.9314
GFP	222	<0.0001	2.166	1.614 to 2.906
GR100-GFP	170	Reference Group		
GR100-GFP siRNA Kapβ2	98	0.0001	0.4659	0.3167 to 0.6854

Table 1: statistics referred to Kaplan Meier curves in Figure 3C

Supplementary Table 2	# cells	p-value	Hazard ratio	95% CI
GFP - 400 ng	447	Reference Group		
GFP-TNPO1 - 400 ng	493	0.0714	1.18	0.9857 to 1.413

GFP - 800 ng	151	Reference Group		
GFP-TNPO1 - 800 ng	264	0.3556	1.13	0.8390 to 1.476

Table 2: statistics referred to Kaplan Meier curves in Figure 4B and 6B

Supplementary Table 3	# cells	p-value	Hazard ratio	95% CI
mCherry	811	<0.0001	0.6796	0.5882 to 0.7853
mCherry GR50	722	Reference Group		
mCherry GR50/Kapβ2 OE	520	<0.0001	0.6494	0.5538 to 0.7615

	# cells	p-value	Hazard ratio	95% CI
mCherry	811	<0.0001	0.726	0.6242 to 0.8444
mCherry GR100	623	Reference Group		
mCherry GR100/Kapβ2 OE	404	0.0054	0.7791	0.6536 to 0.9287

Table 3: statistics referred to Kaplan Meier curves in Figure 5D and 5E

Table 4

Patient	R/G	Yr of Death	age at DOD	age at onset	ATPSY INTERV	Site of Onset	approx. duration in months	Diagnosis	Notes
103	M	2015	22		9HRS			Ctrl	Metastatic synovial sarcoma
101	F	2015	70		32HRS			Ctrl	Respiratory failure secondary to lung cancer and infection
95	M	2013	72		7HRS			Ctrl	Cardiorespiratory failure; Pneumonia, myodysplastic syndrome, acute renal failure
JWAC 06	C/F	2018	91		1 day			Ctrl	N/A
JWAC 29	C/M	2019	55		2 days			C9orf72	
JWAC 11	C/M	2019	58		1 day			C9orf72	
JWAC 104	C/M	2023	53		1 day	Bulbar		C9orf72	

Table 4: patients demographics
