# Supplementary material for: The nuclear import receptor Kapβ2 modifies neurotoxicity mediated by poly(GR) in C9orf72-linked ALS/FTD
Source: Commun Biol. 2024 Mar 28;7:376. doi: 10.1038/s42003-024-06071-2 (PMC10978903; doi:10.1038/s42003-024-06071-2)
Supplement: Supplementary file 2 — Description of Supplementary Materials [file 42003_2024_6071_MOESM2_ESM.docx]

**Description of Additional Supplementary Files**

**File name:** Supplementary Movie 1

**Description:** Imaris rendering of GFP-GR50 mouse spinal cord stained for Neun (magenta) and Kapβ2 (Red). GFP-GR50 is in green

**File name:** Supplementary Movie 2

**Description:** Imaris rendering of a single neuron over expressing Kapβ2. Kapβ2 is in green and nuclei are in blue
