# Supplementary material for: The nuclear import receptor Kapβ2 modifies neurotoxicity mediated by poly(GR) in C9orf72-linked ALS/FTD
Source: Commun Biol. 2024 Mar 28;7:376. doi: 10.1038/s42003-024-06071-2 (PMC10978903; doi:10.1038/s42003-024-06071-2)
Supplement: Supplementary file 5 — Reporting Summary [file 42003_2024_6071_MOESM5_ESM.pdf]

nature portfolio

Corresponding author(s): Davide Trotti, PhD.

Last updated by author(s): Dec 17, 2023

Reporting Summary

Nature Portfolio wishes to improve the reproducibility of the work that we publish. This form provides structure for consistency and transparency in reporting. For further information on Nature Portfolio policies, see our Editorial Policies and the Editorial Policy Checklist.

Statistics

For all statistical analyses, confirm that the following items are present in the figure legend, table legend, main text, or Methods section.

n/a	Confirmed	
☐	☒	The exact sample size (*n*) for each experimental group/condition, given as a discrete number and unit of measurement
☐	☒	A statement on whether measurements were taken from distinct samples or whether the same sample was measured repeatedly
☐	☒	The statistical test(s) used AND whether they are one- or two-sided<br>*Only common tests should be described solely by name; describe more complex techniques in the Methods section.*
☒	☐	A description of all covariates tested
☐	☒	A description of any assumptions or corrections, such as tests of normality and adjustment for multiple comparisons
☐	☒	A full description of the statistical parameters including central tendency (e.g. means) or other basic estimates (e.g. regression coefficient) AND variation (e.g. standard deviation) or associated estimates of uncertainty (e.g. confidence intervals)
☐	☒	For null hypothesis testing, the test statistic (e.g. *F*, *t*, *r*) with confidence intervals, effect sizes, degrees of freedom and *P* value noted<br>*Give P values as exact values whenever suitable.*
☒	☐	For Bayesian analysis, information on the choice of priors and Markov chain Monte Carlo settings
☒	☐	For hierarchical and complex designs, identification of the appropriate level for tests and full reporting of outcomes
☒	☐	Estimates of effect sizes (e.g. Cohen's *d*, Pearson's *r*), indicating how they were calculated

Our web collection on statistics for biologists contains articles on many of the points above.

Software and code

Policy information about availability of computer code

Data collection	No software nor custom code were used for data collection
Data analysis	Data Analysis were performed using RStudio 2022.07.2 through custom made code

For manuscripts utilizing custom algorithms or software that are central to the research but not yet described in published literature, software must be made available to editors and reviewers. We strongly encourage code deposition in a community repository (e.g. GitHub). See the Nature Portfolio guidelines for submitting code & software for further information.

Data

Policy information about availability of data

All manuscripts must include a data availability statement. This statement should provide the following information, where applicable:

- Accession codes, unique identifiers, or web links for publicly available datasets
- A description of any restrictions on data availability
- For clinical datasets or third party data, please ensure that the statement adheres to our policy

The data that support the findings of this study are not openly available due to reasons of sensitivity and are available from the corresponding author upon reasonable request. Data are located in controlled access data storage at Thomas Jefferson University.

Human research participants

Policy information about studies involving human research participants and Sex and Gender in Research.

Reporting on sex and gender	N/A
Population characteristics	N/A
Recruitment	N/A
Ethics oversight	N/A

Note that full information on the approval of the study protocol must also be provided in the manuscript.

Field-specific reporting

Please select the one below that is the best fit for your research. If you are not sure, read the appropriate sections before making your selection.

☒ Life sciences ☐ Behavioural & social sciences ☐ Ecological, evolutionary & environmental sciences

For a reference copy of the document with all sections, see nature.com/documents/nr-reporting-summary-flat.pdf

Life sciences study design

All studies must disclose on these points even when the disclosure is negative.

Sample size	In animal studies, data were gathered from three or more animals. In the case of in vitro experiments, data were collected from a minimum of two distinct experiments, and analysis consistently involved more than three neurons per experiment.
Data exclusions	no data were excluded from the analysis
Replication	data were replicated
Randomization	N/A
Blinding	N/A

Reporting for specific materials, systems and methods

We require information from authors about some types of materials, experimental systems and methods used in many studies. Here, indicate whether each material, system or method listed is relevant to your study. If you are not sure if a list item applies to your research, read the appropriate section before selecting a response.

Materials & experimental systems

n/a	Involved in the study	
☐	☒	Antibodies
☐	☒	Eukaryotic cell lines
☒	☐	Palaeontology and archaeology
☐	☒	Animals and other organisms
☒	☐	Clinical data
☒	☐	Dual use research of concern

Methods

n/a	Involved in the study	
☒	☐	ChIP-seq
☒	☐	Flow cytometry
☒	☐	MRI-based neuroimaging

Antibodies

Antibodies used	anti-Kapb2 (RRID:AB_2206884, Santa Cruz Biotechnology, Inc Cat.# sc-101539; WB dilution 1:1000); anti-Kapb2 (RRID:AB_262123, Sigma Cat.# T0825; IF dilution 1:500); anti-Map2 (RRID:AB_2138178 Novus Cat.#NB300-213; IF dilution 1:2000); anti-GFP (RRID:AB_11042881, Proteintech Cat.#50430-2-AP, WB dilution 1:1000); anti-TDP-43 (RRID:AB_615042, Proteintech Cat.#10782-2-AP; IF dilution 1:500); anti-GAPDH (RRID:AB_2107436, Proteintech Cat.#60004-1-Ig; WB dilution 1:1000); Neun (D4G40) XP(RRID:AB_2651140, Cell Signaling Technology Cat.#24307; IF dilution 1:500): FUS (RRID:AB_2247082, Proteintech Cat.#11570-1-AP; IF:1:500)<br>HRP-Conjugated anti-rabbit (RRID:AB_772191, Cytiva Cat.# NA9340-1ML; WB dilution 1:10,000); HRP-Conjugated anti-mouse

(RRID:AB_772193, Cytiva Cat.# NA9310-1ML; WB dilution 1:10,000); HRP-Conjugated anti-rat (RRID:AB_2936877, Sigma Cat.# AP136P; WB dilution 1:3,000);
AlexaFluor-546 anti-mouse (RRID:AB_2534012, Thermo Fisher Scientific Cat.# A10036; IF dilution 1:1,000); AlexaFluor-647 anti-rabbit (RRID:AB_2536183, Thermo Fisher Scientific Cat.# A31573; IF dilution 1:1,000); AlexaFluor-647 anti-chicken (RRID:AB_2762845, Thermo Fisher Scientific Cat.# A32933; IF dilution 1:1,000);

Validation	*Describe the validation of each primary antibody for the species and application, noting any validation statements on the manufacturer's website, relevant citations, antibody profiles in online databases, or data provided in the manuscript.*

Eukaryotic cell lines

Policy information about cell lines and Sex and Gender in Research

Cell line source(s)	HEK 293 FT cells used for lentivirus production were purchased from Thermo Scientific (cat # R70007)
Authentication	N/A;
Mycoplasma contamination	All cell lines used in this study tested negative for mycoplasma contamination
Commonly misidentified lines (See ICLAC register)	N/A

Animals and other research organisms

Policy information about studies involving animals; ARRIVE guidelines recommended for reporting animal research, and Sex and Gender in Research

Laboratory animals	B6.C-Tg(CMV-cre)1Cgn/J::B6.C-GR50-GFP<br>B6.C-Tg(CMV-cre)1Cgn/J::B6.C-GFP
Wild animals	N/A
Reporting on sex	The gender of the animals in all experiments was not disclosed, as there were no observed sex differences concerning the study's phenotypes.
Field-collected samples	N/A
Ethics oversight	N/A

Note that full information on the approval of the study protocol must also be provided in the manuscript.
